# Comprehensive verbal fluency features predict executive function performance

**DOI:** 10.1038/s41598-021-85981-1

**Published:** 2021-03-25

**Authors:** Julia Amunts, Julia A. Camilleri, Simon B. Eickhoff, Kaustubh R. Patil, Stefan Heim, Georg G. von Polier, Susanne Weis

**Affiliations:** 1 Institute of Neuroscience and Medicine (INM-7 Brain and Behaviour), Forschungszentrum Jülich, Wilhelm-Johnen-Str, 52428 Jülich, Germany; 2 Institute of Systems Neuroscience, Heinrich-Heine University, Moorenstr. 5, 40225 Düsseldorf, Germany; 3 Institute of Neuroscience and Medicine (INM-1 Structural and functional organization of the brain), Forschungszentrum Jülich, Wilhelm-Johnen-Str, 52428 Jülich, Germany; 4 Department of Psychiatry, Psychotherapy und Psychosomatics, Medical Faculty, RWTH Aachen University, Pauwelsstraße 30, 52074 Aachen, Germany; 5 Department of Child and Adolescent Psychiatry, Psychosomatics and Psychotherapy, Goethe-Universität Frankfurt am Main, Deutschordenstraße 50, 60528 Frankfurt am Main, Germany; 6 Department of Child and Adolescent Psychiatry, Psychosomatics and Psychotherapy, Medical Faculty, RWTH Aachen University, Neuenhofer Weg 21, 52074 Aachen, Germany

**Keywords:** Human behaviour, Language

## Abstract

Semantic verbal fluency (sVF) tasks are commonly used in clinical diagnostic batteries as well as in a research context. When performing sVF tasks to assess executive functions (EFs) the sum of correctly produced words is the main measure. Although previous research indicates potentially better insights into EF performance by the use of finer grained sVF information, this has not yet been objectively evaluated. To investigate the potential of employing a finer grained sVF feature set to predict EF performance, healthy monolingual German speaking participants (n = 230) were tested with a comprehensive EF test battery and sVF tasks, from which features including sum scores, error types, speech breaks and semantic relatedness were extracted. A machine learning method was applied to predict EF scores from sVF features in previously unseen subjects. To investigate the predictive power of the advanced sVF feature set, we compared it to the commonly used sum score analysis. Results revealed that 8 / 14 EF tests were predicted significantly using the comprehensive sVF feature set, which outperformed sum scores particularly in predicting cognitive flexibility and inhibitory processes. These findings highlight the predictive potential of a comprehensive evaluation of sVF tasks which might be used as diagnostic screening of EFs.

## Introduction

Executive functions (EFs) comprise cognitive processes that enable goal directed behaviour^1^. Previous literature investigated the general cognitive processes that fall under the umbrella term of EFs and encompass both lower-level cognitive processes and higher-level processes. The former include working memory, inhibition and cognitive flexibility which represent the building blocks for higher-level processes such as planning, reasoning and problem solving^2^.

While the number and definition of different EF subprocesses remains controversial^3^, there is strong evidence that EFs are impaired in a large number of neurological^4,5^ and psychiatric^6,7^ diseases. Therefore, the measurement of EFs forms a crucial part of the clinical neuropsychological diagnostical routine in order to detect and specify impairments such as frontal lobe damages^8^. Multiple test batteries such as the *Delis-Kaplan Executive Function System* (D-KEFS)^9^ and the *Vienna Test System*^10^ provide numerous EF tests to capture a wide range of the different aspects of EFs. However, many EF tests are mainly based on pen-and-paper versions which tend to be time consuming while also lacking accuracy. Moreover, there are discrepancies between unnatural test instructions and naturalistic tasks in everyday life which leads to a lack of ecological validity of commonly used EF tests^11^.

There is consensus, that EFs play a crucial role in speech production processes^12,13^. Cognitive flexibility is required to activate general lexical concepts while later working memory capacities are needed for remembering already produced words. Here, the episodic buffer and phonological loop, which are also related to the working memory system, serve as central components^12^. Since EFs are also involved in speech production, verbal fluency (VF) tests are integrated in several clinical diagnostic batteries to assess EFs. E.g. in *B-CATS*—an assessment tool for schizophrenia; *NIH stroke scale* – assessment for quantifying stroke severity; BCSB—screening for mild Alzheimer´s disease; and D-KEFS—broadly applicable assessment used for assessing diseases such as epilepsy or Parkinson´s disease.

Two different types of VF tests are commonly used. On the one hand, VF is assessed with a lexical task. In this task participants are asked to produce as many words as possible with a specific initial letter within a specific timeframe (usually 1–2 min). Due to the fact that all requested words start with the same phoneme, the lexical VF task is also commonly referred to as *phonological* VF task. On the other hand, the semantic VF (sVF) task requires the production of words belonging to a specific category (e.g. animals or fruits), regardless of the initial letter of the word. The lexical VF task is driven by phonological and lexical cues, whereas the sVF task requires attributes of a specific semantic category.

Within each type of the VF task, it is also possible to modulate the demand on EFs by applying a switching component. Here, participants are asked to switch between two different categories in alternating order within the same task (e.g. fruits-jobs). VF performance is generally evaluated by calculating the total number of correctly produced items. However, in the neurological literature, it has been shown that specific patterns of VF impairment greatly depend on the damaged brain regions^14,15^. Thus, studies suggest the need for a more differentiated analysis of VF performance^16^.

In general, there is consensus on the involvement of EFs in the VF task in healthy controls^17^ as well as their impairment in patients^15,18^. In detail, it is assumed that semantic knowledge and memory as well as cognitive flexibility are required to build semantic associations in sVF tasks whereas the lexical VF tasks require the suppression of grouping words with shared associations^19^. Additionally, in both types of VF tasks, inhibition is presumably needed to suppress competitive responses and to avoid perseveration errors^20,21^, while attention, updating and working memory processes are simultaneously involved to keep the processing speed high, to remember already produced items and to produce as many items as possible^12^.

Although previous findings undergirded the involvement of executive control processes in the VF task^22^, the diagnostic validity of VF tasks to assess EF performance remains controversial^23,24^. In particular, it has been found to be affected by multiple factors such as the underlying language component in the VF task, underlying cognitive processes such as intelligence, and fluctuating hormonal levels^25,26^. Moreover, the literature is not in agreement with regards to the specific relationship between VF and EF. Various studies report a positive correlation between working memory, inhibition, cognitive flexibility performance and the total score of produced words^22,27^. In contrast, other studies failed to identify a clear relationship between VF performance and EFs in one or more EF domains^24,28^. Notably, in previous studies, classical statistic methods were used to e.g. investigate group comparisons of EF performance in patients and healthy controls. Applying correlational analyses, studies investigated linear relationships of VF sum scores and different EF domains^29^.

However, within the last years VF tasks per se have gained more interest as a predictive tool for clinical decision making, e.g. in schizophrenia^30^ or mild cognitive impairments^31^ since they offer an alternative to the highly time-consuming testing procedure of EFs^11^. The growing interest in the predictive value of VF tasks might be a result of the increasing use of machine learning algorithms investigating speech production to predict disease specific properties^32–34^. The main appeal of the machine learning approach is its ability to train a predictive model by identifying patterns in high dimensional data which can be subsequently used to make predictions in unseen data. Additionally, interpreting models can provide information with regards to which specific features contribute most to accurate predictions. Based on a data-driven learning, predictive modelling enables researchers to capture (non)-linear relationships, generalize associations and to potentially subsequently transfer these to a clinical context.

Although the VF task is commonly evaluated based on the total sum of correct produced words^35–37^, other variables can also be employed to gain deeper insights into cognitive performance.

Recent studies have demonstrated the potential of advanced parameters taken from the VF task, i.e. error types^38^, latencies^39^ and semantic distances^18^ to complement the common analysis of the total sum of words. These additional variables, assessed within the sVF task, were shown to reflect the complex involvement of executive processes in disorders such as dementia as well as in better differentiation between patients and healthy controls^16,40^.

To interpret VF performance in more detail, studies have also investigated error types that occurred in the course of the VF task such as those based on the breaking of sVF-specific rules (e.g. naming words from a different category, creating neologisms) and category errors. Perseveration and category errors are particularly reported in the switching VF task when participants fail to switch to the second category, name words from a different category or repeat the same category twice^37^. Thus, perseveration and category errors can provide qualitative information when measuring VF performance, in addition to the commonly used total sum of words.

Additionally, information of the VF task can also be assessed on a semantic level, analysing semantic relatedness of produced words. This concept was first investigated by Troyer et al.^41^ who manually organized produced words in the sVF task into conceptually related clusters and switches. Specifically, semantically related words were clustered based on specific subcategories^41^. For example, animals were clustered based on their living environment, human use and zoological categories. According to these clusters, which are usually defined as a minimum of a two-word-sequence within the same subcategory, switches were calculated as the total number of shifts between these clusters^41^. Here, two types of switches were defined: While *cluster switches* describe a transition between multiword and adjacent clusters, *hard switches* represent transitions between a cluster and non-clustered words^42^. Later research showed that the ability to create new subcategories and generate new cues is more important for performing the sVF task than creating large cluster sizes^43^. Moreover, authors highlighted the importance of working memory capacity for self-generating category cues in healthy participants^43^ and suggested the sVF task as a diagnostic tool in cognitive impairment^44,45^. Nevertheless, this assessment of semantic information from the sVF task was traditionally done manually and thus was highly time-consuming and partially subjective due to the manual determination and assignment of subcategories^46,47^. However, this problem can be addressed with the help of computational linguistics providing automated computational approaches (e.g. Latent semantic analysis^48^, Word2Vec^49^). Nowadays large text corpora and fine grained information of semantic relatedness are available (e.g. WordNet^50^, DISCO^51^). In general, different conceptual structures are implemented in these models. On the one hand, some systems provide the hierarchical structure of a lexical semantic net^52^ based on semantic concepts (e.g. fishes, birds, mammals)^50^. In contrast to this hierarchical and ontological approach, vector-based systems rely on the co-occurrence of words within a big text corpus. Here, words are represented as a point in a multi-dimensional space creating word embeddings^53^. Applying these computerized and automated systems, studies were able to identify dementia risk in healthy participants based on semantic relatedness^16^ and to distinguish between patients with forms of disorganization and healthy controls^54^.

Alongside the semantic information, the sVF task also provides prosodic information such as speech latencies (speech pauses between each word). Latencies convey information about the approximate time needed to access lexical items^13,55^. Although there is little literature on the relationship between speech latencies in the VF task and EF performance, some findings indicate that it might be meaningful^39,43^. Specifically, studies suggest that a higher incidence of unfilled pauses are more likely to occur in situations in which participants are confronted with a higher planning load^56^. Other studies also report a relationship between prosodic information and EF demands showing a decreased production of words within the progress of the VF task^39^. Since a decrease of the number of produced words in the VF task also indicates an increase of speech latencies^39^ these findings suggest that speech latencies could provide additional information on VF performance with respect to the involvement of EFs.

In summary, previous studies indicate the potential of additional quantitative measures for evaluating sVF performance to gain better insight into cognitive processes. However, diagnostic batteries used in the clinical context as well as in the scientific environment still heavily rely on the sole use of the sum of correct words as the main indicator of EF performance. Consequently, the aim of the present study was to investigate the predictive power of a comprehensive set of sVF measures and compare it to the commonly used sum score analysis. As a first step into deeper insights of the predictive power of the VF task, we focus on the semantic VF task which allowed us to exploit the vast information within the semantic relatedness features. In this exploratory study, machine learning methods were applied to predict performances of well validated but highly time-consuming EF tests from a broad set of objective and mainly computerized VF measurements in unseen participants. We expected the extended sVF feature set to outperform the basic analysis of sum scores in predicting EF test results.

## Methods

### Participants

In this study, 230 healthy participants with an age range of 20–55 years (mean age 35.2 ± 11.1; 92 males) were tested. Before the actual testing session, participants were asked for previously detected diagnoses. Only participants without neurological or psychiatric diagnoses were included in this study. Moreover, participants were monolingual German speakers, i.e. their native language was German and they did not learn an additional language before going to school. Participants received different levels of education (finished middle school: 8, professional school/job training: 63, finished high school with a university-entrance diploma: 69, university degree: 90). The recruitment took place in North Rhine-Westphalia (Germany) via social networks and the Forschungszentrum Jülich mailing list. Participants were tested at the Forschungszentrum Jülich, and the testing session included an EF test battery together with VF tasks, with a duration of 150–180 min depending on the individual time needed for instructions and the speed with which the participants passed the tests. A remuneration fee of €50 was paid. All experiments were performed in accordance with relevant guidelines and regulations. Moreover, informed consent was obtained from all participants. Collection and analyses of the data presented here was approved by the ethics committee at Heinrich-Heine University Düsseldorf.

### Executive function assessment

The EF test battery consisted of 14 computerized versions of commonly used neuropsychological tests covering domains of cognitive flexibility, working memory and inhibition. While 11 of these tests were taken from the *Vienna Testsystem*^10^, three were designed with *PsyToolkit*^57^. The *Vienna Testsystem*^10^ is a standardized computerized test battery providing numerous EF tests and test manuals. Every EF test provided multiple variables which were extracted automatically by the respective test system. While some of these variables represent main variables, others solely include processing time information which are not directly linked to the EF performance. EF tests which were designed within *PsyToolkit*^57^ do not come with associated test manuals and the selection of variables of these tests was thus based on previous literature^58–60^.

Cognitive flexibility was assessed using five tests, namely, the *Trail Making Test*^61^ (TMT), *Raven´s Standard Progressive Matrices*^62^ (SPM), *Wisconsin Card Sorting Test*^63^ (WCST), *Tower of London* (TOL)^64^ and *Cued-Task Switching*^65^ (SWITCH).

Working memory performance was examined using three tests: *N-back non-verbal Test*^66^ (NBN), *Non-verbal Learning Test*^67^ (NVLT) and *Corsi Block Tapping Test*^68^ (CORSI).

Inhibition was tested using *Stop-Signal Task*^69^ (STOP), *Simon Task*^70^ (SIMON) and *Stroop Test*^71^ (STROOP).

Additionally, we also assessed divided and spatial attention (WAF-G^72^, WAF-R^72^) as well as vigilance (*Mackworth Clocktest*^60^ (CLOCK)). In total, 68 variables were extracted from EF tests. The full set of EF test variables is provided in the supplementary material (Table S1).

### Semantic verbal fluency tasks

The sVF tasks were based on the *Regensburger Wortflüssigkeitstest*^37^ (RWT) which is equivalent to the English *Controlled Oral Word Association Test*^73^ (COWAT). The German standardized neuropsychological version of the VF task was used due to language-specific differences in the frequency and usage of letters and categories^36^. Two of the tasks were simple sVF tasks in which the participant had to name animals (t_1_) and jobs (t_2_). The third sVF task (t_3_) was a switching task in which the participant switched between fruits and sports within the same task. Each of the three tasks was performed for 2 min. The sVF tasks were presented with *Presentation* software^74^ and the participant´s responses were recorded automatically.

Following the testing session, the recorded speech was transcribed and words were coded manually as being either *correct answers* or *errors*. Furthermore, errors were differentiated into perseveration and category errors. Sum scores of each sVF tasks separately, sum score of correct produced words across all sVF and errors (perseveration, category errors) were included in the prediction analysis. In general, the sum scores solely include correct produced items in all three sVF tasks. A list of extracted sVF features is shown in Table 1.

**Table 1:** Overview of Verbal fluency features.

VF features	Description
Correct words t1 + t2 + t3	Sum of all correct produced words in task1, task2, task3
Correct words	Sum of correct produced words in each task
Switch coefficient	Relationship of correct items in simple and switching tasks; switching coefficient = sum3/((sum1 + sum2)/2))
Repetition error	Repetition errors in task 1, task 2
Category error	Category errors in task 3
Latency mean	Mean of speech breaks in each task
Latencies 1st quarter	Mean of speech breaks in seconds 0–30 (i1) for each task
Latencies 2nd quarter	Mean of speech breaks in seconds 31–60 (i2) for each task
Latencies 3rd quarter	Mean of speech breaks in seconds 61–90 (i3) for each task
Latencies 4th quarter	Mean of speech breaks in seconds 91–120 (i4) for each task
Latency difference	Progress of speech breaks (i4-i1) in each task
Sequential mean	Semantic mean of all sequential word pairs in each task; computed with GermaNet (hierachical)
Cumulative mean	Semantic mean of all possible word pairs (cumulative) in each task; computed with GermaNet (hierarchical)
Sequential mean cat1 t3	Semantic mean of all sequential word pairs (sequential) in catergory 1 (sports) of switching task; computed with GermaNet (hierarchical)
Sequential mean cat2 t3	Semantic mean of all sequential word pairs (sequential) in catergory 2 (fruits) of switching task; computed with GermaNet (hierarchical)
Sequential mean DIS	Semantic mean of all sequential word pairs in each task; computed with DISCO (Word2Vec)
Cumulative mean DIS	Cumulative mean of all possible word pairs in each task; computed with DISCO (Word2Vec)
Sequential mean cat1 t3 DIS	Semantic mean of all sequential word pairs in category 1 (sports) of switching task; computed with DISCO (Word2Vec)
Sequential mean cat2 t3 DIS	Semantic mean of all sequential word pairs in category 2 (fruits) of switching task; computed with DISCO (Word2Vec)

Speech latencies were automatically detected and manually corrected using *PRAAT*^75^, and the mean of the speech *latencies* within each task was calculated. Moreover, the task was divided into four 30-seconds intervals (i_1_, i_2_, i_3_, i_4_) and the mean of the speech latencies within each interval was determined. Additionally, these means of intervals were then used to determine an increase or decrease of speech latencies within each task (i_4_-i_1_). Latency means of each task and of each interval as well as latency differences were defined as sVF features for prediction analysis.

*Semantic distances* were computed using two different approaches to ensure that the results of prediction analysis are not dependent on a specific semantic system. One of the semantic systems was a hierarchical structured lexical-semantic net of *GermaNet*^52^ and *GermaNet-Pathfinder*^76^. Specifically, this lexical network is partitioned into various sets of semantic concepts (*synsets*) that are intertwined by semantic relations and create nodes. These synsets are related conceptually in different ways including, hypernymy, part-whole relations, entailment and causation^52^, leading to hierarchical-structured subcategories. *GermaNet-Pathfinder*^76^ provides different measurements^77^ for the determination of how closely two nouns are related to each other. In this study, we selected a path-based measure which describes the relatedness between concepts. In detail, the path-based system takes the distance between two synset nodes and the longest possible shortest path between any two nodes in GermaNet into account.

$$sim\left({s}_{1},{s}_{2}\right)= \frac{MAXSHORTESTPATH-length({s}_{1},{s}_{2})}{MAXSHORTESTPATH}$$

length(s_1_,s_2_) = shortest path between synset s1 and synset s2.

MAXSHORTESTPATH = maximum of all shortest paths within GermaNet.

Applying this formula, semantic relatedness is represented by values between 0 and 1. While closely related words lead to values approximating 1 (German Shepard x Labrador → sim = 0.94), more distanced word pairs lead to smaller values (e.g. German Shepard x dolphin → sim = 0.77).

The other semantic system that was used to determine semantic similarity between words was *DISCO*^78^ applying a Word2Vec^49^ approach. This system is based on co-occurrences in large text corpora. Specifically, this corpus contains 1.5 billion tokens including German Wikipedia entries, newspaper articles, parliamentary debates, movie subtitles and more. Each unique word is represented by a word vector and is part of the vector space. Within this vector space, word vectors are located based on shared common contexts building word embeddings. As in *GermaNet*^52^, a high semantic similarity is represented by numbers approximating 1.

Each sVF task of the participants was analysed automatically using *GermaNet Pathfinder*^76^ and *DISCO API*^78^. For our feature-set which was later used for the prediction analysis, two different types of semantic relations were extracted: (1) Sequential distance was computed across each consecutive word pair in order of the produced words. (2) Cumulative distance was computed over the entire task regardless of the order in which they appear within the task. As an output, the relatedness between each word-pair was extracted and the mean of all semantic relations within one task was calculated. In the case that *GermaNet* contained more than one synset for one word, the synset with closest relatedness to the paired word was selected. Moreover, missing lexical entries in *GermaNet* or *DISCO* led to a deletion of the corresponding word pair. All semantic information, including means of sequential and cumulative distances of both systems (*GermaNet* and *DISCO*) were added as features to prediction analysis.

Altogether, 43 features were extracted from the sVF tasks containing information of sum of correct words, error types, speech latencies and semantic distances calculated with two different systems. A complete overview of VF feature scores is provided in the supplementary material (Table S2).

### Machine learning analysis

In this study, we applied a machine approach using a cross-validation procedure. Here, just parts of the data are used to train the model while the other part is used to validate the model; i.e. EF scores were predicted in unseen participants which allows for generalization of results to a certain degree.

EF performance was predicted from sVF variables (*features*) applying supervised learning via random forests^79,80^ (RF). The sVF features were used to predict each of the 68 EF scores (*targets*) in separate and independent analyses. Generally speaking, RF creates a “forest” of decision trees as weak learners by randomly sampling the features before learning each decision tree. The trees are used as an ensemble and the prediction of individual trees is averaged to get the final prediction^81^. In the present study 100 trees were used to compute prediction analysis.

Previous work indicates that performance in the VF task is negatively related to age^82,83^. Moreover, sex was found to be associated with differential solving strategies in the VF task^84^. Likewise, a higher level of education was associated with better performance in VF tasks^82,85^. Therefore, data was transformed to z-scores and sex, age and education were regressed out from the sVF features within cross-validation. A tenfold cross-validation procedure was performed for which the data set was randomly split into 10 sets, 9 of which were used for training while the 10th set was held back and used to assess the prediction performance in previously unseen data. Ten repetitions of the tenfold cross-validation were performed and thus 100 prediction models for each EF target were computed. Prediction performance was assessed by computing the mean correlation (*Pearson*) between real and predicted values within cross-validation folds and subsequently across all repetitions. EF targets which were predicted from sVF features at a significance level of *p* < 0.01 were considered *highly predictable* EF targets.

To compare the predictive power of the comprehensive and the classical feature set, the prediction analysis was computed for classical sVF features, solely containing information from sum scores of sVF tasks.

The sVF features which contributed most strongly to the prediction analyses of each *highly predictable* EF target were identified. Feature importance was defined by the permutation of out-of-bag predictor observations as implemented in *Matlab*^86^. The top five sVF features with the highest feature performance were identified to further investigate the (non) linear relationship of these sVF features with the respective EF performance. Here, rank correlations (*Spearman*) of sVF features and EF test scores were calculated. Due to the high number of extracted EF variables, only one highly significantly predicted EF test variable of each EF test is presented to exemplarily demonstrate the complex relationship of sVF features and EF performance. The selection of this representative EF variable was based on the test EF manuals and previous literature describing specific main variables of each EF test.

## Results

### Prediction of EF variables from verbal fluency data

**Figure 1:**
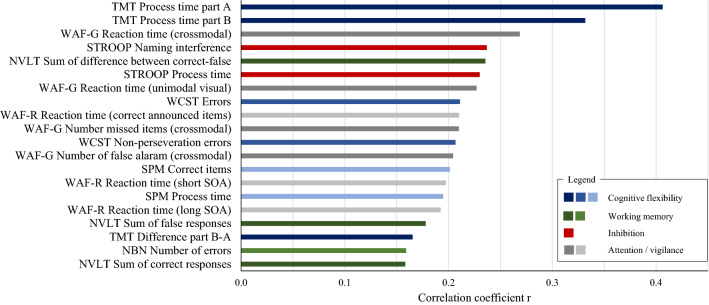
Correlation coefficients of true and predicted executive function variables computed with full feature set. Executive function variables were predicted based on 43 verbal fluency features. Results shown in this table illustrate executive function variables which could be predicted at p < 0.01 from verbal fluency data; Colour groups indicate EF domains and colour gradients denote different EF tests within this EF domain; *NBN* N-back non-verbal; *NVLT* Non-verbal learning test; *SOA* Stimulus onset asynchrony; *SPM* Raven’s standard progressive matrices; *STROOP* Stroop test; *TMT* Trail making test; *WCST* Wisconsin Card Sorting Test; *WAF-G* Divided attention; *WAF-R* Spatial attention.

**Figure 2:**
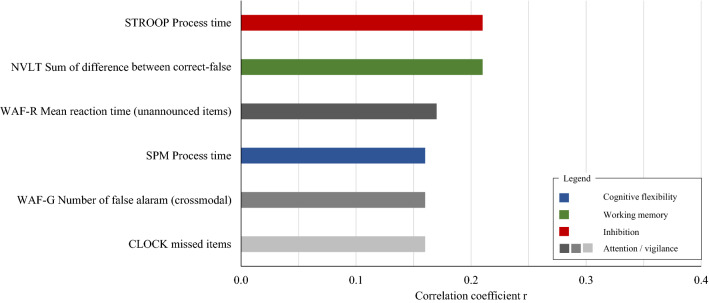
Correlation coefficients of true and predicted executive function variables computed with classical feature set. Executive function variables were predicted based on the sum scores of all 3 semantic verbal fluency tests as well as the total sum score across these 3 tests, which led to a total number of four verbal fluency features. Results shown in this table illustrate executive function variables which could be predicted with p < 0.01 from verbal fluency data; Colour groups indicate EF domains and gradients denote different EF tests within this EF domain; *MACK* Mackworth Clock Test; *NVLT* non-verbal learning test; *SPM* Raven’s Standard Progressive Matrices; *STROOP* Stroop Test; *WAF-G* Divided attention; *WAF-R* Spatial attention.

To investigate which EF targets were predictable from sVF features, we computed two independent prediction analyses. In the first analyses the full set of sVF features, including sum scores, errors, latencies and semantic relatedness was used (Fig. 1). The second analysis was performed with variables containing only information regarding the number of correctly produced items in each sVF task (Fig. 2). Both figures show the EF targets that were significantly predicted from sVF features at a significance level of *p* < 0.01. Detailed results of all prediction analyses are given in the supplementary material (Table S3).

In sum, 20 EF targets, pertaining to 8 different EF tests and tapping into all subdomains of EFs, could be predicted significantly from the full feature set. With respect to cognitive flexibility, TMT, SPM and WCST were predicted from sVF data. The highest correlation between true and predicted values was identified in processing times of part A (r = 0.41; *p* = 3.2e^−10^) and B (r = 0.33; *p* = 2.6e^−7^) of TMT. While these results are primarily related to overall processing speed, an explicit relationship between sVF performance and cognitive flexibility was found in the “*difference between part B-A*” of the TMT (r = 0.17 *p* = 0.007) as well as in the test results of SPM and WCST. Here, the number of correct items in the SPM (r = 0.20; *p* = 0.001) and different error types in the WCST revealed the complexity of cognitive requirements and planning ability in conducting the sVF task. With regards to tests assessing working memory capacity, two of three EF tests, namely NVLT (r = 0.24; *p* = 0.0002) and NBN (r = 0.16; *p* = 0.009) were predicted significantly. Beside EF targets referring to cognitive flexibility and working memory, the analysis also identified inhibition targets. Particularly, *naming interference* (r = 0.24; *p* = 0.0002) and processing time in STROOP (r = 0.23; *p* = 0.0003) were predicted.

Across all subdomains of EFs, variables displaying general processing speed and reaction times performance were detected. The role of attention and general processing speed is also represented in test results referring to divided and spatial attention. Here, seven targets of attention tests were predicted significantly. In general, tests from all EF subdomains were predicted significantly and no dominance of one specific subdomain was apparent.

The focus of this study was the predictive power of an advanced VF feature set. To compare the predictive power of the advanced features with the commonly used VF information, i.e. the sum of correctly produced words, an additional prediction analysis was computed using solely sum scores. Here, the sum scores of each sVF tasks as well as a total sum score across all three tests were included. In this analysis only six EF targets were predicted significantly (Fig. 2). Prediction performance was lower than in the analysis with full feature set and particularly targets of processing speed and reactions times were detected. In contrast to the first analysis, vigilance was predicted with missed items in CLOCK (r = 0.16; *p* = 0.009).

### Impact of sVF features on prediction analysis

The impact of single sVF features on EF performance was quantified based on the feature importance scores of the prediction analysis. Due to the high number of significantly predicted EF targets, only one EF target for each of the significantly predicted EF tests is discussed in detail here. We focus on the main variables for the respective EF tests based on previous literature and the EF test manuals. For each of these, the five most important sVF features were extracted and correlations with the respective EF target were calculated (Tables 2, 3, 4, 5) to enable a comparison of present results with commonly used univariate analyses.

**Table 2:** Spearman correlations of five most important semantic verbal fluency (sVF) features with significantly predictable cognitive flexibility targets.

SPM—correct items				TMT—difference part B-A				WCST—non-perseveration errors			
Top 5 sVF features		r	p	Top 5 sVF features		r	p	Top 5 sVF features		r	p
1	Repetition error t_1_	− 0.05	0.44	1	Latencies 4th quarter t_1_	− 0.01	0.88	1	Repetition error t_3_	− 0.10	0.12
2	Latencies 2nd quarter t_1_	− 0.09	0.18	2	**Repetition error t**_**3**_	**− 0.12**	**0.08**	2	**Latencies 1st quarter t**_**1**_	**0.17**	0.01*
3	Category error t_3_	− 0.01	0.87	3	Latency difference t_1_	− 0.02	0.80	3	Category error t_3_	0.01	0.85
4	**Correct words t**_**1**_	**− 0.12**	**0.08**	4	Category error t_3_	− 0.07	0.27	4	**Correct words t**_**2**_	**− 0.16**	0.02*
5	**Cum. mean t**_**3**_	**0.14**	0.05*	5	Repetition error t_1_	− 0.01	0.80	5	**Total sum score t**_**1**_** + t**_**2**_** + t**_**3**_	**− 0.16**	0.02*

1–5 = Top five sVF features with regards to predictor performance based on feature importance; correlations with *p* < 0.1 are marked in bold; significant correlations (*p* < 0.05) are marked with *.

*SPM* Raven’s Standard Progressive Matrices; *TMT* Trail-Making Test; *WCST* Wisconsin Card Sorting Test.

t_1_ = VF test (animals); t_2_ = VF test (jobs); t_3_ = Switching VF test (sports/fruits); *Cum* cumulative.

**Table 3:** Spearman correlations of five most important semantic verbal fluency (sVF) features with significantly predictable working memory targets.

NBN—errors				NVLT—difference correct minus errors			
Top 5 sVF features		r	p	Top 5 sVF features		r	P
1	Repetition error t_3_	− 0.01	0.91	1	**Latencies 4th quarter t**_**1**_	**− 0.12**	**0.08**
2	**Sequ. mean t**_**1**_	**− 0.19**	0.00*	2	Category error t_3_	− 0.03	0.63
3	Category error t_3_	− 0.01	0.92	3	**Latency difference t**_**1**_	**− 0.12**	**0.08**
4	Repetition error t_1_	0.11	0.11	4	Correct words t_1_	0.07	0.28
5	**Cum. mean DIS t**_**3**_	**− 0.12**	**0.07**	5	Latency mean t_2_	0.03	0.69

1–5 = Top five VF features with regards to predictor performance based on feature importance; correlations with *p* < 0.1 are marked in bold; significant correlations (*p* < 0.05) are marked with *.

*NBN* N-back non-verbal; *NVLT* non-verbal learning test.

t_1_ = VF test (animals); t_2_ = VF test (jobs); t_3_ = Switching VF test (sports/fruits); *Cum* cumulative; *Sequ* sequential; *DIS* semantic system *DISCO.*

**Table 4:** Spearman correlations of five most important semantic verbal fluency (sVF) features with significantly predictable inhibition target.

STROOP—naming interference			
Top 5 sVF features		r	p
1	**Cum. mean t**_**2**_	**− 0.18**	0.01*
2	Latency difference t_1_	0.03	0.62
3	Latencies 4th quarter t_1_	0.05	0.48
4	**Sequ. mean DIS cat**_**1**_** t**_**3**_	**− 0.15**	0.03*
5	**Total sum score t**_**1**_** + t**_**2**_** + t**_**3**_	**− 0.22**	0.00*

1–5 = Top five VF features with regards to predictor performance based on feature importance; correlations with *p* < 0.1 are marked in bold; significant correlations (*p* < 0.05) are marked with *.

t_1_ = VF test (animals); t_2_ = VF test (jobs); t_3_ = Switching VF test (sports/fruits); *Cum* cumulative; *Sequ* sequential; *DIS* semantic system *DISCO.*

**Table 5:** Spearman correlations of five most important semantic verbal fluency (sVF) features with significantly predictable attention targets.

WAF-G reaction time crossmodal				WAF-R—reaction time correctly announced			
Top 5 sVF features		r	P	Top 5 sVF features		r	p
1	Repetition error t_3_	− 0.09	0.17	1	Repetition error t_2_	− 0.01	0.93
2	Repetition error t_2_	− 0.03	0.70	2	Repetition error t_1_	− 0.09	0.17
3	**Latencies 1st quarter t**_**3**_	**0.13**	**0.06**	3	Sequ. mean DIS t_1_	0.04	0.61
4	Repetition error t_1_	− 0.05	0.45	4	Cum. mean DIS t_1_	0.07	0.29
5	Cum. Mean t_1_	− 0.03	0.59	5	Repetition error t_3_	0.01	0.88

1–5 = Top five VF features with regards to predictor performance based on feature importance; correlations with *p* < 0.1 are marked in bold; significant correlations (*p* < 0.05) are marked with *.

*WAF-G* divided attention test; *WAF-R* spatial attention test.

t_1_ = VF test (animals); t_2_ = VF test (jobs); t_3_ = Switching VF test (sports/fruits); *Cum* cumulative; *Sequ* sequential; *DIS* semantic system *DISCO.*

Across all EF domains, the most important sVF features for the prediction results included information about number of correctly produced words, error types, latencies and semantic distances. Out of these most predictive sVF features, some showed a significant correlation with the EF target (*p* < 0.05), while others displayed a trend level significance (p < 0.1) or no significant correlation at all. In the following, we assessed the top five sVF features that are related to the different EF subdomains of cognitive flexibility, working memory, inhibition as well as to attention. Due to the high number of EF scores that were predicted significantly from sVF features, one EF variable of each significantly predicted test is presented here. A complete overview of the correlation matrix of all sVF features and significantly predicted EF scores is given in the supplementary material (Tables S4–S6).

With regards to cognitive flexibility (Table 2) 7/15 sVF features were related to errors participants produced within the sVF task. Repetition errors in simple and switching sVF tasks as well as category errors in the switching task were found to be important sVF features for predicting EF targets. Particularly, repetition and category errors were determined as highly relevant in predicting *TMT* performance. However, no significant (linear) correlation between errors and cognitive flexibility performance was found. In contrast, a linear relationship of sVF information and EF performance was shown for the number of correctly produced words. Here, significant correlations of correctly produced words and cognitive flexibility targets were primarily found in the *WCST*. Similar but not significant results were also found in the *SPM*. In all three significantly predicted EF tests (*SPM, TMT, WCST*) latencies within the sVF task_1_ (animals) were identified as important sVF features but did not reveal correlations with EF targets except for latency patterns assessed in i_1_. Here, longer speech breaks were shown to positively correlate with errors in WCST. With regards to semantic relatedness the cumulative mean within the sVF switching task (t_3_), calculated with the hierarchical structured approach of *GermaNet*, was identified as a meaningful feature predicting *SPM* performance. Specifically, participants naming closely related words across both switching categories (sports and fruits) achieved better *SPM* targets.

Within the EF domain of working memory, the *NBN* and *NVLT* were identified as highly predictable EF tests (Table 3). Here, the sum of correctly produced words was selected as an important sVF feature less often than for cognitive flexibility tests and no significant correlation with EF target was found. Non-linear relationships of sVF features and working memory performance was additionally found for sVF features *errors* which were mainly important for predicting *NBN* performance. Among the five most important sVF features predicting NBN performance, the sequential as well as cumulative mean of the semantic relatedness were found to be highly relevant. Similar to results in cognitive flexibility tests (Table 1), a smaller search space (r = − 0.12 *p* = 0.07) and closely related words (r = − 0.19 *p* = 0.005) led to better results in *NBN*. While semantic relatedness was particularly important for predicting errors in *NBN*, latencies were relevant for *NVLT* performance. Here, results indicated a relationship between smaller speech breaks in end of the sVF task and higher NVLT target (r = − 0.12 *p* = 0.08).

With respect to inhibition, naming interference in the *Stroop* test was predicted significantly. While error types were not selected as most important sVF features, the total sum score across all three sVF tests was determined as meaningful and revealed a significant correlation with *Stroop* performance (r = − 0.22 *p* < 0.001) (Table 4). Important features for predicting *naming interference* performance were semantic relatedness and latencies. In particular, the searching space in t_2_ represented by the cumulative mean was identified as highly important. These results indicate a better inhibition performance if participants searched for less distanced words (r = − 0.18 *p* = 0.01). Similar results were also found in sVF features of sequential relatedness. Searching for closely related words in the first category within the switching sVF task (cat_1_ t_3_) was related to better inhibitory performance (r = − 0.15 *p* = 0.03). Beside sematic relatedness and total sVF sum score, the analysis also points toward the relevance of latency patterns within the first sVF task (animals) for predicting inhibitory processes.

Finally, we investigated sVF features in the prediction of attentional performance (Table 5). Here, the results demonstrate a predictive importance of repetition errors in simple as well as in switching sVF tasks. The results revealed no significant correlation between number of errors and attention performance. Latencies within the first quarter of the switching sVF task (t_3_) were selected as relevant for attention performance, indicating that a higher processing speed in the beginning of the sVF task resulted in faster reaction times in the divided attention test. Similar to previously reported results in other EF subdomains, semantic relatedness features in simple sVF task (animals) were selected as meaningful variables for attention performance.

To sum up, across all subdomains of EFs, a variety of different types of sVF features, including sum scores, error types, sematic relatedness and latencies showed high relevance for the prediction of EF performance. Out of these, about one third showed significant or trend level correlation with EF targets, while the remaining VF features that were identified as important for prediction accuracy, did not show any linear relationship with the respective EF target.

## Discussion

### Main findings

This study aimed to investigate whether EF performance can be predicted from sVF tasks using Machine Learning methods. In a first step, we applied a RF approach to determine which EF tests could successfully be predicted from a wide range of VF information. Results of this machine learning analysis identified EF tests tapping into all subdomains of EFs. In total, 20 of 44 EF scores were predicted significantly when using the full set of sVF features which included errors, latencies and semantic distances.

Moreover, prediction results of the full sVF features set was compared to a classical feature set including only sum scores of sVF tasks, as commonly used in clinical settings. The comparison of these two approaches revealed a larger number of significantly predicted EF scores as well as higher prediction accuracy of the advanced feature set. Particularly for cognitive flexibility performance, the comprehensive feature set achieved a higher prediction accuracy as compared to the commonly used sum score evaluation. Thus, the present results clearly demonstrate the advantage of using more comprehensive sVF features over the sole use of sum scores, which to date still tend to be the most common measure used to asses sVF tasks. In a second step, we further investigated the concrete involvement of different types of sVF features to gain insights into the impact of specific VF aspects on EF performance. Results showed that all types of sVF features, i.e. sum scores, errors, latencies and semantic relatedness contributed to the prediction of EF. With regards to the different EF subdomains no dominance of specific VF types was detected. Moreover, the correlation analyses revealed that good sVF predictors do not necessarily correlate with the respective EF score.

The following section starts with a discussion of the influence of different sVF features on prediction results. Here, predictable EF tests within each subdomain are presented and the contributions of sVF features are interpreted. Additionally, the role of general processing speed is addressed. Secondly, advantages of an elaborated VF feature set are delineated. In the end, limitations of this study are considered.

### Sum scores

Summarizing scores of correctly produced items is the most commonly used way of evaluating VF tasks in the clinical and scientific context to date. The present study included separate sum scores for each sVF test as well as a total one across all sVF tasks. Results revealed the importance of sum score features for the prediction of cognitive flexibility, working memory and inhibition performance. In contrast, sum scores were not identified as important for predicting attention scores. Particularly sum scores resulting from t_1_ (animals) as well as total sum scores revealed high feature importance. Furthermore, a positive linear relationship of relevant sVF sum scores and EF performance in the domains of inhibition and cognitive flexibility was found.

The findings from the present study can be directly linked to previous studies. In particular, Paula et al.^87^ reported a positive correlation between cognitive flexibility performance, assessed with the TMT, and the sum of correct produced words in the switching task. With regards to working memory, another study found an association between the sum of correct produced items and working memory performance^43^. With respect to inhibition, our findings are also in line with multiple studies that demonstrated the positive linear relationship of inhibition performance and the total sum of words, assessed within the VF task, both in older^22^ and young^88^ adults.

Overall, based on previous literature and the results of the current study, sum scores were shown to contribute to the prediction results. In accordance with previous findings, this contribution appears to be based on a positive linear relationship of sum scores with EF performance.

### Error types

When predicting EF test scores from sVF features, repetition and category errors were identified to mainly contribute to the prediction of cognitive flexibility, working memory and attention test result. Conversely, errors were not identified as important features for the prediction of inhibition scores. While both repetition and category errors were shown to be equally important for the prediction of cognitive flexibility and working memory, only repetition errors contributed to predicting attention performance. Importantly, in contrast to sum scores, most error features did not show a linear relationship with the respective EF test performance. The prediction results of TMT were the only ones to reveal a correlation trend, indicating that fewer repetition errors in sVF tasks are associated with better cognitive flexibility performance.

These findings partially contradict previous findings investigating the linear relationship between errors in the VF task and EF performance. Particularly, previous studies suggested that executive inhibitory dysfunction and reduced working memory performance lead to a higher number of perseveration errors in healthy participants^20,89^. Similar findings have also been reported in patients with brain damages^90^ and schizophrenia^38^. In contrast, some studies did not find an increase in the number of perseveration errors in Parkinson´s patients compared to healthy controls^91^.

Although in the present study repetition and category errors were shown to be important for the successful prediction of EF performance in all EF domains except for inhibitory processes, results revealed that a low number of produced errors does not necessarily result in better EF performance. Due to the importance of errors in prediction results and the non-linear relationship with EF performance, we assume that some participants adopt strategies where a higher number of errors is accepted in order to achieve a better score in the sVF task. Thus, successful EF performance does not necessarily go along with fewer errors.

### Latencies

With regards to latency patterns our results revealed the importance of speech breaks for the prediction of all domains of EF as well as for attention scores. Latency patterns contributed differently to the prediction of different EF scores. Latency patterns during the first interval of the sVF task (i_1_) were revealed as a meaningful feature for inhibitory processes, cognitive flexibility and attention performance. However, additional latency patterns, such as the mean of all latencies within each task and the progress of latencies (namely *latency differences*) also contributed to the prediction results. Interestingly, our results indicate an ambiguous relationship between latency patterns and EF test results. On the one hand, correlation analyses revealed some significant correlations between latency patterns and EF scores with, for example, longer speech breaks in i_1_ were related to a higher amount of errors in the WCST assessing cognitive flexibility performance. On the other hand, most of the latency features did not show a linear relationship with EF performance.

To our knowledge, the relationship between speech breaks and EF performance in the context of VF has rarely been reported in previous literature, with existing studies tending to rather focus on unfilled pauses in free speech^56^. However, previous findings support our results with respect to the importance of speech breaks within the first interval of the VF task in that previous studies found a relationship between longer latencies in the beginning of the VF task and cognitive flexibility performance^39^. Moreover, other studies suggest that a decrease of speech latencies over the course of the VF task is related to the cluster patterns of the participants. While participants are assumed to produce clusters with high-frequent words in the beginning of the task, less frequent words are produced during the progression of the task leading to more switches and increased searching times^92^.

In general, previous studies support the positive relationship between the duration of speech breaks and higher cognitive demands^56^. However, our results revealed mostly non-linear relationships between latencies and significantly predicted EF scores. This might suggest that shorter speech breaks per se do not go along with better EF performance. Rather, we assume that the heterogeneity of searching strategies, including processes such as clustering and switching, lead to ambiguous latency patterns.

### Semantic relatedness

Investigating the role of semantic relatedness between produced words within the sVF task, two different semantic analysis systems were applied. On the one hand, a hierarchical approach was used (*GermaNet*)^52^. On the other hand, an approach based on word embeddings was applied (DISCO)^51^. The main goal of including both approaches was to assess as much diverse semantic information as possible. Our results revealed that semantic relatedness measures from both semantic systems contribute essentially to the prediction of all EF domains as well as to attention performance. Although not all semantic features revealed a linear relationship with EF performance, results indicate that searching for closely related words might be related to stronger EF test results.

These findings are partially in line with previous studies which apply earlier approaches of cluster and switching quantification to investigate the importance of switches in the sVF task^87^. Authors have found a positive relationship between fewer switches and better cognitive flexibility performance in healthy participants^87^. In contrast, other studies reported a decreased number of switches in depressive patients with reduced cognitive flexibility^93^. Although the present study did not differentiate between the two types of switches^42^, the semantic systems applied in this study^51,52^ provided additional semantic distances which are similarly interpretable. In detail, these semantic measurements also quantify semantic distances of sequential and cumulative word pairs. Thus, a higher semantic mean in the present study can be equated to a higher cluster size and less hard switches. However, the present study did not aim to investigate such a fine-grained semantic approach as Troyer´s^41^ approach but rather strived to investigate the general importance of semantic distances within the sVF task.

In general, we assume that the production of semantically distanced words puts higher demands on cognitive processes. However, for the sVF task, participants are asked to simply produce as many words as possible, with no demands on the number of different subcategories these words come from. Thus, producing closely related words and building high cluster sizes might represent the most efficient strategy of successful EF performers.

### Superiority of advanced sVF feature set

While the full feature set of sum scores, errors, latencies and semantic relatedness was applied for the main analysis, we also predicted EF scores using sum score features only. Using the sophisticated feature set, test variables from all EF domains as well as attention performance and 8/14 EF tests were successfully predicted. While many of the predictable EF scores contained general information of processing speed and reaction times, results also comprised EF scores which are considered as characteristic variables for specific EF tests. For example, TMT is represented by the *difference between part A-B*^94^, Stroop by *naming interference*^71^, SPM by *correct items*^62^ and WCST by *non-perseveration errors*^58^*,* all of which were found to be predictable EF scores.

In contrast, analysis with a classical sVF feature solely containing information of the sum scores, predicted only 6/14 EF tests most of which were related to general processing speed rather than to specific EF functions. Only one EF score of NVLT contained characteristic information of working memory performance. EF scores representing cognitive flexibility and inhibitory performance did not include information which are directly linked to EF performance but rather related to general speed.

To our knowledge, so far, no other study has attempted combining different types of sVF measurements to predict EF scores. However, previous research has demonstrated the advantages of advanced approaches evaluating additional information over the sole use of the total number of correctly produced words. For example, it was shown that the switching sVF task, which was also used in the present study (t_3_), contained more information of cognitive flexibility than simple VF tasks^87^. Our findings are also in line with another study investigating the digitalized evaluation of semantic relatedness with WordNet^50^. In particular, semantic relatedness was found to be highly associated with EFs and serve as an indicator for mild cognitive impairments which are difficult to detect with sum scores^95^.

The comparison of prediction analysis with and without an extended set of sVF features mainly indicated that sum scores alone capture mostly working memory performance and attention scores. On the other hand, an advanced sVF feature set including sum scores, errors, latencies and sematic relatedness allows for the prediction of cognitive flexibility, working memory and inhibition performances as well as attention scores.

Investigating the relationship between the most important sVF features and EF performance in more detail, multiple non-linear relationships were detected. These findings highlight the advantages of machine learning approaches which are able to detect complex, non-linear relationships in addition to straightforward linear ones. Also, these approaches can take into account multivariate interactions between different VF features to reveal patterns which could not have been identified based on each single feature alone.

In general, our findings indicate that the use of a comprehensive set of VF features might have the potential to replace time-consuming and artificial EF tests. Due to the use of abstract symbols like numbers and letters, commonly used neuropsychological tests are criticized for their lacking ecological validity^11^. In contrast, producing words which are related to a specific category better represents daily needs and requirements of participants. Moreover, the lack of ecological validity might have influenced the correlations of the abstract EF test scores and the more natural sVF features. However, it remains open whether comprehensive sVF features may be even more helpful in clinical practice than commonly used EF test batteries.

### Role of processing speed

In both analyses, variables which are not directly linked to EF performance but rather represent overall processing speed or reaction times, were predicted significantly. Similar findings were reported in our previous study predicting VF sum scores from EF tests variables^96^. The relationship of processing speed and sVF performance is also reported in other studies^83,97^. These authors suggest that processing time reflects general cognitive abilities such as intelligence to some extent^98^ but may also be related to age^99^ or personality traits such as extraversion^100^. Additionally, the presence of a time indication within some EF tests might facilitate processing speed similarly as in sVF tasks.

### Limitations

Our results yielded insights into the involvement of EFs in the sVF task and highlighted the informative value of the sVF task to predict EF performance using a comprehensive feature set. Moreover, our results revealed complex and mostly non-linear relationships of VF features and EF performance. Hence, a detailed examination of individual differences in searching strategies might improve our understanding of which sVF patterns are related to higher EF performance in certain domains. As with all analyses of individual differences such research is dependent on large data sets comprising detailed information on EF and VF performance.

An additional consideration relates to the generalizability of our results. Ideally, our findings should be validated in a fully independent data set. To date, such a data set of sufficient size is not yet available. Hence, we applied a cross-validation approach within our sample. Here, the model was trained on some parts of the data while other parts of the data were held back. The model was then validated in the previously held back participants. This within-dataset validation represents the best alternative when a fully independently acquired dataset is not yet available.

### Summary and outlook

Our study revealed insights into the advantages of an elaborated analysis of sVF tasks which successfully predicts EF performance. In comparison to the commonly used approach of evaluating sum scores of correctly produced words, we detected a lucid advantage of an extended feature analysis. In particular with regards to cognitive flexibility and inhibition our study demonstrated that an evaluation of sVF sum scores does not capture actual EF performance but rather assesses overall processing speed. Thus, we suggest the utilization of a comprehensive analysis of VF performance including features of error types, latencies and semantic distances. The present study applied primarily automated and digitalized methods ensuring a time-efficient and objective evaluation of VF performance. Further studies ought to develop a fully automated software tool integrating and further developing our feature set. Here, it would be highly interesting to also include features from the lexical VF task. A computerized toolbox allowing for an extensive assessment of VF could serve as a screening tool for EFs in a clinical diagnostic process as well as in a research context. Such a tool could include an audio system that records the speech of the patient and converts it into text. Subsequently, an automated software could be used to automatically determine a comprehensive set of VF features including sum scores, errors, latencies and semantic distances from the transcribed data. This can in turn result in a digitalized and quantified evaluation of the patient´s EFs compared to healthy controls based on VF performance, which can be then used by the clinician as part of the diagnostic process. Consequently, this toolbox could allow for higher ecological validity while also saving time in clinical routine.

However, we do not suggest that VF assessments will be able to fully substitute an initial extensive assessment of EFs with commonly used EF test batteries. We rather propose an extended and fully digitalized VF analysis as part of progress diagnostics in the form of a screening to assess EF performance in e.g. Parkinson´s disease or ADHD. Additionally, this screening-tool could be used in patients with predispositions of schizophrenia before manifestation of clinical symptoms. Here, an advanced sVF analysis could provide insights into subtle changes of EF performance. In the future, this work might contribute to an automated digitalized speech analysis supporting clinicians in diagnostic processes.

Altogether, the present study demonstrated the predictive superiority of an extended VF feature evaluation. Additionally, the results provided a first step towards an automated analysis of VF serving as a predictor for EFs.

## Supplementary Information

Supplementary Information.
