# Supplementary material for: Comprehensive verbal fluency features predict executive function performance
Source: Sci Rep. 2021 Mar 25;11:6929. doi: 10.1038/s41598-021-85981-1 (PMC7994566; doi:10.1038/s41598-021-85981-1)
Supplement: Supplementary file 1 — Supplementary Information. [file 41598_2021_85981_MOESM1_ESM.pdf]

# Comprehensive verbal fluency features predict executive function performance

Julia Amunts*[1,2], Julia A. Camilleri[1,2], Simon B. Eickhoff[1,2], Kaustubh R. Patil[1,2], Stefan Heim[3,4], Georg von Polier[1,5,6] & Susanne Weis[1,2]

[1] Institute of Neuroscience and Medicine (INM-7 Brain and Behaviour), Forschungszentrum Jülich, Germany
[2] Institute of Systems Neuroscience, Heinrich-Heine University, Düsseldorf, Germany
[3] Institute of Neuroscience and Medicine (INM-1 Structural and functional organization of the brain), Forschungszentrum Jülich, Germany
[4] Department of Psychiatry, Psychotherapy and Psychosomatics, Medical Faculty, RWTH Aachen University, Aachen, Germany
[5] Department of Child and Adolescent Psychiatry, Psychosomatics and Psychotherapy, Goethe-Universität Frankfurt am Main, Frankfurt am Main, Germany
[6] Department of Child and Adolescent Psychiatry, Psychosomatics and Psychotherapy, Medical Faculty, RWTH Aachen University, Aachen, Germany

Corresponding author:
Julia Amunts
Institute of Neuroscience and Medicine (INM-7 Brain and Behaviour), Research Center Jülich, Jülich, Germany
Wilhelm-Johnen-Str.
52428 Jülich
GERMANY

Correspondence to j.amunts@fz-juelich.de

## Supplementary Table S1: Test variables of executive function tests

| Test | Abbreviation | Variable | Mean | SD |
|---|---|---|---|---|
| | COGNITIVE FLEXIBILITY | | | |
| Trail Making test | TMT | Process time partA | 17.11 | 3.17 |
| | | Process time partB | 24.70 | 7.51 |
| | | Diference part B-A [seconds] | 7.59 | 6.30 |
| | | Quotient B/A | 1.45 | 0.36 |
| | | Errors part A | 0.06 | 0.28 |
| | | Errors part B | 0.65 | 1.00 |
| Raven´s Standard Progressive Matrices | SPM | Correct items | 27.82 | 3.31 |
| | | process time | 629.56 | 157.94 |
| Wisconsin Card Sorting Test | WCST | Number of errors | 12.68 | 6.17 |
| | | Number of perseveration errors | 7.96 | 3.49 |
| | | Number of errors (non perseveration) | 4.72 | 3.29 |
| | | Timeouts | 0.52 | 1.05 |
| Tower of London | TOL | Planning ability | 7.52 | 2.19 |
| | | Number of correct responses | 10.52 | 1.61 |
| | | Changed his/her mind, self-correction | 1.82 | 2.08 |
| | | Choice of wrong pole | 0.50 | 1.16 |
| | | Choice of blocked pole | 1.03 | 2.11 |
| | | Choice of impossible position | 0.79 | 2.34 |
| Cued Task Switching | SWITCH | Number of errors | 2.53 | 3.34 |
| | | Switch costs (reaction time switch tasks - reaction time in non-switch tasks) [seconds] | 0.05 | 0.08 |
| | | Timeouts | 0.28 | 0.78 |
| | | Errors of items which are incongruent | 2.06 | 2.41 |
| | WORKING MEMORY | | | |
| N-back non verbal | NBN | Correct items | 8.36 | 2.94 |
| | | Number of commission errors | 5.64 | 2.94 |
| | | Number of errors | 8.86 | 7.89 |
| | | Mean reaction tine of correct items [seconds] | 0.78 | 0.17 |
| | | Mean reaction time of errors [seconds] | 0.87 | 0.24 |
| Non-verbal learning test | NVLT | Sum of correct responses | 32.09 | 5.03 |
| | | Sum of false responses | 12.57 | 7.93 |
| | | Sum of difference between correct minus false responses | 19.52 | 7.85 |
| | | Process time | 119.16 | 33.97 |
| Corsi Block Tapping Test | CORSI | Block span | 5.66 | 1.09 |
| | | Correct items | 10.59 | 2.81 |
| | | False items | 4.59 | 1.31 |
| | | Missed items | 0.04 | 0.19 |
| | | Sequency errors | 2.59 | 1.27 |
| | INHIBITION | | | |
| Stop Signal Task | INHIB | Reaction time [seconds] | 0.51 | 0.09 |
| | | Mean stop signal delay [seconds] | 0.30 | 0.06 |
| | | Stop signal reaction time [seconds] | 0.21 | 0.07 |
| | | Number of commission errors | 14.37 | 6.20 |
| | | Number of ommission errors | 1.29 | 1.83 |
| Simon Task | SIMON | Reaction time difference (reaction time incongruent - reaction time congruent items) [seconds] | 0.03 | 0.04 |
| | | Number of errors in compatible items | 2.85 | 11.17 |
| | | Number of errors in incompatible items | 5.15 | 12.46 |
| Stroop Test | STROOP | Reading interference [seconds] | 0.14 | 0.08 |
| | | Naming interference [seconds] | 0.13 | 0.08 |
| | | Interference-difference [seconds] | -0.05 | 0.12 |
| | | Number of false reactions (reading-baseline) | 2.14 | 2.52 |
| | | Number of false reactions (naming-baseline) | 2.67 | 2.47 |
| | | Number of false reactions (reading-interference) | 3.36 | 6.60 |
| | | Number of false reactions (naming-interference) | 3.52 | 3.19 |
| | | Process time | 404.96 | 61.21 |
| | ATTENTION / VIGILANCE | | | |
| Divided attention test | WAF-G | Number of missed items (unimodall visual) | 2.08 | 3.10 |
| | | Number of false alarm (unimodal visual) | 3.30 | 3.49 |
| | | Mean reaction time (unimodal visual) [ms] | 445.70 | 89.16 |
| | | Number of missed items (crossmodal visual/auditive) | 2.76 | 2.95 |
| | | Number of false alarm (crossmodal visual/auditive) | 3.10 | 4.90 |
| | | Mean reaction time (crossmodal) [ms] | 457.16 | 110.11 |
| Spatial attention test | WAF-R | Mean reaction time (unannounced items) [ms] | 379.84 | 47.69 |
| | | Number of missed items (correct announced items) | 0.95 | 1.55 |
| | | Mean reaction time (correct announced items) [ms] | 306.51 | 47.97 |
| | | Number of missed items (wrong announced items) | 0.21 | 0.55 |
| | | Mean reaction time (wrong announced items) [ms] | 339.46 | 52.46 |
| | | Mean reaction time (short SOA) [ms] | 347.63 | 44.23 |
| | | Mean reaction time (long SOA) [ms] | 341.17 | 44.24 |
| | | Number of errors | 3.58 | 3.34 |
| Mackworth Clock Test | MACK | Number of missed jumps | 7.88 | 4.93 |
| | | Number of false alarms | 3.15 | 3.78 |

## Supplementary Table S2: Overview of verbal fluency task results

| Variable | Description | Mean | SD |
|---|---|---|---|
| Correct words t1+t2+t3 | Sum of all correct produced words in t1+t2+t3 | 84.83 | 15.54 |
| | Verbal fluency task 1 (animals) | | |
| Correct words t1 | Sum of correct produced words in task 1 | 36.73 | 8.24 |
| Repetition error t1 | Repetition errors in task 1 | 0.60 | 0.89 |
| Latency mean t1 | Mean of speech breaks in task 1 | 2.88 | 0.86 |
| Latencies 1st quarter t1 | Mean of speech breaks in seconds 0-30 (i1) | 1.41 | 0.48 |
| Latencies 2nd quarter t1 | Mean of speech breaks in seconds 31-60 (i2) | 3.20 | 1.94 |
| Latencies 3rd quarter t1 | Mean of speech breaks in seconds 61-90 (i3) | 4.23 | 2.71 |
| Latencies 4th quarter t1 | Mean of speech breaks in seconds 91-120 (i4) | 6.48 | 6.51 |
| Latency difference t1 | Progress of speech breaks (i4-i1) in task 1 | 5.07 | 6.50 |
| Sequential mean t1 | Semantic mean of all sequential word pairs in task 1; computed with GermaNet (hierachical) | 0.83 | 0.02 |
| Cumulative mean t1 | Semantic mean of all possible word pairs (cumulative) in task 1; computed with GermaNet (hierarchical) | 0.80 | 0.04 |
| Sequential mean t1 DIS | Semantic mean of all sequential word pairs in task 1; computed with DISCO (Word2Vec) | 0.45 | 0.05 |
| Cumulative mean t1 DIS | Cumulative mean of all sequential word pairs in task 1; computed with DISCO (Word2Vec) | 0.36 | 0.03 |
| | Verbal fluency task 2 (jobs) | | |
| Correct words t2 | Sum of correct produced words in task 2 | 26.08 | 6.65 |
| Repetition error t2 | Repetition errors in task 2 | 0.39 | 0.75 |
| Latency mean t2 | Mean of speech breaks in task 2 | 3.91 | 1.30 |
| Latencies 1st quarter t2 | Mean of speech breaks in seconds 0-30 (i1) | 2.17 | 0.80 |
| Latencies 2nd quarter t2 | Mean of speech breaks in seconds 31-60 (i2) | 4.06 | 1.95 |
| Latencies 3rd quarter t2 | Mean of speech breaks in seconds 61-90 (i3) | 6.32 | 5.16 |
| Latencies 4th quarter t2 | Mean of speech breaks in seconds 91-120 (i4) | 6.85 | 6.75 |
| Latency difference t2 | Progress of speech breaks (i4-i1) in task 2 | 4.68 | 6.69 |
| Sequential mean t2 | Semantic mean of all sequential word pairs in task 2; computed with GermaNet (hierarchical) | 0.85 | 0.02 |
| Cumulative mean t2 | Semantic mean of all possible word pairs (cumulative) in task 2; computed with GermaNet (hierarchical) | 0.82 | 0.02 |
| Sequential mean t2 DIS | Semantic mean of all sequential word pairs in task 2; computed with DISCO (Word2Vec) | 0.39 | 0.08 |
| Cumulative mean t2 DIS | Semantic mean of all possible word pairs (cumulative) in task 2; computed with DISCO (Word2Vec) | 0.27 | 0.03 |
| | Verbal fluency task 3 (sports / fruits) | | |
| Correct words t3 | Sum of correct produced words in task 3 | 22.01 | 4.38 |
| Repetition error t3 | Repetition errors in task 3 | 0.20 | 0.48 |
| Category error t3 | Category errors in task 3 | 0.10 | 0.39 |
| Latency mean t3 | Mean of speech breaks in task 3 | 4.64 | 1.27 |
| Latencies 1st quarter t3 | Mean of speech breaks in seconds 0-30 (i1) | 2.29 | 0.66 |
| Latencies 2nd quarter t3 | Mean of speech breaks in seconds 31-60 (i2) | 5.16 | 2.15 |
| Latencies 3rd quarter t3 | Mean of speech breaks in seconds 61-90 (i3) | 7.15 | 4.15 |
| Latencies 4th quarter t3 | Mean of speech breaks in seconds 91-120 (i4) | 9.33 | 8.64 |
| Latency difference t3 | Progress of speech breaks (i4-i1) in task 3 | 7.04 | 8.59 |
| Sequential mean t3 | Semantic mean of all sequential word pairs in task 3; computed with GermaNet (hierarchical) | 0.60 | 0.02 |
| Cumulative mean t3 | Semantic mean of all possible word pairs (cumulative) in task 3; computed with GermaNet (hierarchical) | 0.74 | 0.02 |
| Sequential mean t3 DIS | Semantic mean of all sequential word pairs in task 3; computed with DISCO (Word2Vec) | 0.08 | 0.08 |
| Cumulative mean t3 DIS | Semantic mean of all possible word pairs (cumulative) in task 3; computed with DISCO (Word2Vec) | 0.23 | 0.02 |
| Sequential mean cat1 t3 | Semantic mean of all sequential word pairs in category 1 (sports) of switching task; computed with GermaNet (hierarchical) | 0.89 | 0.03 |
| Sequential mean cat2 t3 | Semantic mean of all sequential word pairs in category 2 (fruits) of switching task; computed with GermaNet (hierarchical) | 0.90 | 0.02 |
| Sequential mean cat1 t3 DIS | Semantic mean of all sequential word pairs in category 1 (sports) of switching task; computed with DISCO (Word2Vec) | 0.40 | 0.07 |
| Sequential mean cat2 t3 DIS | Semantic mean of all sequential word pairs in category 2 (fruits) of switching task; computed with DISCO (Word2Vec) | 0.52 | 0.05 |
| Switch coefficient | relationship of simple and switching tasks - switching coefficient sum3/((sum1+sum2)/2)) | -9.40 | 5.78 |
| Correct words t1+t2+t3 | Sum of all correct produced words in t1+t2+t3 | 84.83 | 15.54 |

## Supplementary Table S3: Prediction results of executive function targets

| Test | Abbreviation | Variable | Full feature set r | Full feature set p | Reduced feature set r | Reduced feature set p |
|---|---|---|---|---|---|---|
| | COGNITIVE FLEXIBILITY | | | | | |
| Trail Making test | TMT | Process time partA | 0.41 | **0.000** | 0.11 | 0.050 |
| | | Process time partB | 0.33 | **0.000** | 0.14 | 0.018 |
| | | Diference part B-A [seconds] | 0.17 | **0.007** | 0.09 | 0.083 |
| | | Quotient B/A | 0.11 | 0.055 | 0.07 | 0.146 |
| | | Errors part A | 0.00 | 0.487 | 0.05 | 0.209 |
| | | Errors part B | 0.07 | 0.136 | 0.05 | 0.222 |
| Raven´s Standard Progressive Matrices | SPM | Correct items | 0.20 | **0.001** | 0.04 | 0.295 |
| | | process time | 0.19 | **0.002** | 0.16 | **0.007** |
| Wisconsin Card Sorting Test | WCST | Number of errors | 0.21 | **0.001** | 0.06 | 0.198 |
| | | Number of perseveration errors | 0.11 | 0.045 | 0.02 | 0.406 |
| | | Number of errors (non perseveration) | 0.21 | **0.001** | 0.06 | 0.189 |
| | | Timeouts | 0.03 | 0.318 | 0.08 | 0.105 |
| Tower of London | TOL | Planning ability | 0.13 | 0.029 | 0.02 | 0.357 |
| | | Number of correct responses | -0.03 | 0.664 | 0.04 | 0.252 |
| | | Changed his/her mind, self-correction | 0.06 | 0.171 | -0.08 | 0.894 |
| | | Choice of wrong pole | 0.07 | 0.137 | -0.06 | 0.800 |
| | | Choice of blocked pole | 0.00 | 0.526 | 0.01 | 0.432 |
| | | Choice of impossible position | 0.02 | 0.401 | 0.01 | 0.428 |
| Cued Task Switching | SWITCH | Number of errors | 0.11 | 0.055 | -0.06 | 0.826 |
| | | Switch costs (reaction time switch tasks - reaction time in non-switch tasks) [seconds] | 0.03 | 0.345 | 0.00 | 0.505 |
| | | Timeouts | -0.06 | 0.792 | 0.03 | 0.330 |
| | | Errors of items which are incongruent | 0.11 | 0.048 | -0.05 | 0.756 |
| | WORKING MEMORY | | | | | |
| N-back non verbal | NBN | Correct items | 0.08 | 0.135 | 0.01 | 0.422 |
| | | Number of commission errors | 0.08 | 0.121 | 0.00 | 0.497 |
| | | Number of errors | 0.16 | **0.009** | 0.05 | 0.229 |
| | | Mean reaction tine of correct items [seconds] | 0.00 | 0.505 | -0.05 | 0.777 |
| | | Mean reaction time of errors [seconds] | -0.07 | 0.861 | -0.01 | 0.574 |
| Non-verbal learning test | NVLT | Sum of correct responses | 0.16 | **0.010** | -0.03 | 0.660 |
| | | Sum of false responses | 0.18 | **0.004** | 0.07 | 0.163 |
| | | Sum of difference between correct minus false responses | 0.24 | **0.000** | 0.21 | **0.001** |
| | | Process time | 0.01 | 0.452 | 0.15 | 0.011 |
| Corsi Block Tapping Test | CORSI | Block span | 0.12 | 0.039 | -0.05 | 0.771 |
| | | Correct items | 0.15 | 0.012 | -0.07 | 0.846 |
| | | False items | -0.05 | 0.772 | -0.06 | 0.798 |
| | | Missed items | 0.05 | 0.249 | 0.07 | 0.142 |
| | | Sequency errors | -0.04 | 0.728 | 0.05 | 0.220 |
| | INHIBITION | | | | | |
| Stop Signal Task | INHIB | Reaction time [seconds] | 0.15 | 0.010 | 0.12 | 0.037 |
| | | Mean stop signal delay [seconds] | 0.04 | 0.276 | 0.14 | 0.018 |
| | | Stop signal reaction time [seconds] | 0.09 | 0.103 | 0.09 | 0.099 |
| | | Number of commission errors | 0.01 | 0.421 | 0.13 | 0.024 |
| | | Number of ommission errors | 0.09 | 0.100 | 0.03 | 0.327 |
| Simon Task | SIMON | Reaction time difference (reaction time incongruent - reaction time congruent items) [seconds] | 0.08 | 0.126 | -0.12 | 0.970 |
| | | Number of errors in compatible items | 0.00 | 0.505 | -0.03 | 0.689 |
| | | Number of errors in incompatible items | 0.04 | 0.269 | -0.02 | 0.618 |
| Stroop Test | STROOP | Reading interference [seconds] | -0.04 | 0.719 | -0.12 | 0.970 |
| | | Naming interference [seconds] | 0.24 | **0.000** | 0.08 | 0.100 |
| | | Interference-difference [seconds] | 0.13 | 0.024 | -0.02 | 0.596 |
| | | Number of false reactions (reading-baseline) | 0.12 | 0.036 | -0.06 | 0.825 |
| | | Number of false reactions (naming-baseline) | 0.09 | 0.086 | -0.08 | 0.889 |
| | | Number of false reactions (reading-interference) | -0.03 | 0.690 | -0.01 | 0.584 |
| | | Number of false reactions (naming-interference) | 0.12 | 0.043 | -0.08 | 0.897 |
| | | Process time | 0.23 | **0.000** | 0.21 | **0.001** |
| | ATTENTION / VIGILANCE | | | | | |
| Divided attention test | WAF-G | Number of missed items (unimodall visual) | 0.06 | 0.190 | -0.07 | 0.854 |
| | | Number of false alarm (unimodal visual) | 0.04 | 0.291 | 0.03 | 0.353 |
| | | Mean reaction time (unimodal visual) [ms] | 0.23 | **0.000** | 0.10 | 0.061 |
| | | Number of missed items (crossmodal visual/auditive) | 0.21 | **0.001** | 0.16 | **0.008** |
| | | Number of false alarm (crossmodal visual/auditive) | 0.20 | **0.001** | 0.12 | 0.032 |
| | | Mean reaction time (crossmodal) [ms] | 0.27 | **0.000** | 0.08 | 0.128 |
| Spatial attention test | WAF-R | Mean reaction time (unannounced items) [ms] | 0.14 | 0.018 | 0.17 | **0.004** |
| | | Number of missed items (correct announced items) | -0.02 | 0.599 | -0.03 | 0.653 |
| | | Mean reaction time (correct announced items) [ms] | 0.21 | **0.001** | -0.03 | 0.669 |
| | | Number of missed items (wrong announced items) | 0.03 | 0.322 | 0.04 | 0.268 |
| | | Mean reaction time (wrong announced items) [ms] | 0.08 | 0.120 | 0.14 | 0.018 |
| | | Mean reaction time (short SOA) [ms] | 0.20 | **0.002** | 0.07 | 0.139 |
| | | Mean reaction time (long SOA) [ms] | 0.19 | **0.002** | 0.08 | 0.111 |
| | | Number of errors | -0.07 | 0.849 | -0.02 | 0.609 |
| Mackworth Clock Test | MACK | Number of missed jumps | 0.12 | 0.038 | 0.16 | **0.009** |
| | | Number of false alarms | 0.03 | 0.352 | -0.02 | 0.626 |

**Supplementary Table S4: Correlations of all semantic verbal fluency features and significantly predicted EF scores (cognitive flexibility tests)**

| | SPM - Correct items | | SPM - Process time | | TMT - Process time partA | | TMT - Process time partA | | TMT - Difference B-A | | WCST - Errors | | WCST - Non-perseveration errors | |
|---|---|---|---|---|---|---|---|---|---|---|---|---|---|---|
| | r | p | r | p | r | p | r | p | r | p | r | p | r | p |
| Correct words t1+t2+t3 | 0.100 | 0.143 | -0.166 | 0.014 | -0.111 | 0.103 | -0.134 | 0.048 | -0.103 | 0.130 | -0.120 | 0.077 | -0.157 | 0.020 |
| Correct words t1 | 0.118 | 0.081 | -0.069 | 0.312 | -0.142 | 0.036 | -0.092 | 0.176 | -0.038 | 0.579 | -0.086 | 0.207 | -0.112 | 0.100 |
| Correct words t2 | 0.014 | 0.834 | -0.166 | 0.014 | -0.008 | 0.904 | -0.121 | 0.076 | -0.138 | 0.042 | -0.114 | 0.094 | -0.158 | 0.020 |
| Correct words t3 | 0.100 | 0.140 | -0.197 | 0.003 | -0.104 | 0.127 | -0.112 | 0.099 | -0.080 | 0.238 | -0.084 | 0.215 | -0.098 | 0.148 |
| Repetition error t1 | -0.052 | 0.442 | 0.039 | 0.571 | -0.046 | 0.499 | -0.034 | 0.614 | -0.018 | 0.796 | 0.025 | 0.710 | 0.031 | 0.647 |
| Repetition error t2 | -0.044 | 0.522 | -0.028 | 0.680 | -0.064 | 0.346 | -0.046 | 0.501 | -0.022 | 0.746 | 0.027 | 0.688 | 0.017 | 0.800 |
| Repetition error t3 | -0.034 | 0.619 | -0.086 | 0.207 | 0.003 | 0.964 | -0.100 | 0.142 | -0.119 | 0.080 | -0.081 | 0.233 | -0.104 | 0.124 |
| Category error t3 | -0.011 | 0.869 | 0.059 | 0.387 | -0.021 | 0.761 | -0.072 | 0.287 | -0.075 | 0.271 | -0.020 | 0.764 | 0.013 | 0.846 |
| Latency mean t1 | -0.158 | 0.020 | 0.020 | 0.765 | 0.129 | 0.058 | 0.041 | 0.548 | -0.016 | 0.819 | 0.082 | 0.225 | 0.078 | 0.252 |
| Latency mean t2 | -0.022 | 0.746 | 0.110 | 0.105 | -0.022 | 0.746 | 0.082 | 0.230 | 0.107 | 0.115 | 0.092 | 0.175 | 0.106 | 0.117 |
| Latency mean t3 | -0.106 | 0.120 | 0.127 | 0.061 | 0.103 | 0.129 | 0.075 | 0.269 | 0.037 | 0.584 | 0.067 | 0.323 | 0.064 | 0.349 |
| Latencies 1st quarter t1 | -0.130 | 0.055 | 0.101 | 0.138 | 0.129 | 0.057 | 0.130 | 0.054 | 0.089 | 0.188 | 0.211 | 0.002 | 0.165 | 0.014 |
| Latencies 1st quarter t2 | -0.043 | 0.531 | 0.118 | 0.082 | -0.011 | 0.874 | 0.064 | 0.350 | 0.080 | 0.239 | 0.106 | 0.120 | 0.131 | 0.053 |
| Latencies 1st quarter t3 | 0.038 | 0.581 | 0.150 | 0.027 | 0.115 | 0.092 | -0.012 | 0.858 | -0.071 | 0.296 | 0.046 | 0.499 | 0.060 | 0.379 |
| Latencies 2nd quarter t1 | -0.090 | 0.185 | 0.041 | 0.549 | 0.116 | 0.087 | 0.069 | 0.310 | 0.024 | 0.729 | 0.004 | 0.955 | -0.032 | 0.641 |
| Latencies 2nd quarter t2 | 0.009 | 0.898 | 0.121 | 0.074 | 0.001 | 0.986 | 0.078 | 0.249 | 0.092 | 0.178 | 0.053 | 0.438 | 0.093 | 0.171 |
| Latencies 2nd quarter t3 | -0.010 | 0.884 | 0.110 | 0.105 | 0.075 | 0.270 | 0.056 | 0.410 | 0.029 | 0.673 | 0.104 | 0.126 | 0.081 | 0.235 |
| Latencies 3rd quarter t1 | -0.048 | 0.478 | 0.047 | 0.495 | 0.065 | 0.342 | -0.025 | 0.716 | -0.061 | 0.369 | -0.007 | 0.921 | 0.021 | 0.755 |
| Latencies 3rd quarter t2 | 0.040 | 0.560 | 0.078 | 0.253 | -0.010 | 0.883 | 0.038 | 0.572 | 0.050 | 0.461 | 0.003 | 0.960 | -0.018 | 0.796 |
| Latencies 3rd quarter t3 | -0.058 | 0.394 | 0.063 | 0.358 | 0.008 | 0.905 | -0.008 | 0.910 | -0.013 | 0.848 | -0.002 | 0.972 | -0.027 | 0.688 |
| Latencies 4th quarter t1 | -0.139 | 0.041 | -0.075 | 0.272 | 0.089 | 0.188 | 0.029 | 0.672 | -0.010 | 0.879 | 0.048 | 0.478 | 0.107 | 0.114 |
| Latencies 4th quarter t2 | 0.003 | 0.969 | 0.022 | 0.742 | -0.067 | 0.323 | -0.007 | 0.915 | 0.025 | 0.716 | 0.037 | 0.585 | 0.050 | 0.466 |
| Latencies 4th quarter t3 | -0.079 | 0.244 | 0.024 | 0.730 | 0.038 | 0.575 | 0.058 | 0.394 | 0.049 | 0.469 | -0.038 | 0.577 | -0.010 | 0.883 |
| Latency difference t1 | -0.129 | 0.057 | -0.082 | 0.226 | 0.080 | 0.239 | 0.019 | 0.777 | -0.017 | 0.803 | 0.033 | 0.631 | 0.095 | 0.161 |
| Latency difference t2 | 0.008 | 0.911 | 0.009 | 0.898 | -0.066 | 0.329 | -0.015 | 0.828 | 0.016 | 0.819 | 0.025 | 0.714 | 0.035 | 0.612 |
| Latency difference t3 | -0.082 | 0.226 | 0.012 | 0.858 | 0.030 | 0.664 | 0.059 | 0.385 | 0.055 | 0.420 | -0.042 | 0.541 | -0.015 | 0.830 |
| Sequential mean t1 | 0.028 | 0.686 | 0.025 | 0.711 | 0.077 | 0.260 | -0.011 | 0.873 | -0.051 | 0.456 | 0.089 | 0.192 | 0.082 | 0.226 |
| Sequential mean t2 | 0.000 | 0.996 | 0.031 | 0.648 | 0.090 | 0.185 | 0.041 | 0.549 | 0.003 | 0.963 | -0.077 | 0.259 | -0.017 | 0.799 |
| Sequential mean t3 | 0.010 | 0.884 | 0.039 | 0.565 | -0.021 | 0.760 | 0.003 | 0.968 | 0.014 | 0.843 | 0.016 | 0.814 | 0.042 | 0.536 |
| Cumulative mean t1 | 0.093 | 0.172 | 0.021 | 0.759 | 0.031 | 0.646 | 0.058 | 0.398 | 0.052 | 0.444 | 0.007 | 0.921 | 0.082 | 0.231 |
| Cumulative mean t2 | 0.043 | 0.531 | 0.037 | 0.589 | 0.049 | 0.471 | 0.092 | 0.176 | 0.084 | 0.218 | 0.062 | 0.359 | 0.105 | 0.123 |
| Cumulative mean t3 | 0.135 | 0.046 | 0.066 | 0.331 | 0.026 | 0.704 | -0.048 | 0.479 | -0.069 | 0.307 | 0.006 | 0.925 | 0.002 | 0.973 |
| Sequential mean cat1 t3 | 0.100 | 0.141 | 0.074 | 0.276 | -0.045 | 0.513 | -0.055 | 0.416 | -0.043 | 0.528 | -0.071 | 0.299 | -0.029 | 0.674 |
| Sequential mean cat2 t3 | 0.062 | 0.361 | -0.020 | 0.769 | 0.027 | 0.690 | -0.076 | 0.264 | -0.103 | 0.130 | -0.094 | 0.165 | -0.110 | 0.107 |
| Switch coefficient | -0.017 | 0.807 | -0.007 | 0.919 | 0.027 | 0.688 | 0.049 | 0.474 | 0.044 | 0.520 | 0.061 | 0.366 | 0.094 | 0.165 |
| Sequential mean t1 DIS | 0.075 | 0.272 | 0.047 | 0.495 | 0.110 | 0.106 | 0.161 | 0.017 | 0.135 | 0.047 | 0.018 | 0.796 | 0.022 | 0.743 |
| Sequential mean t2 DIS | 0.020 | 0.768 | 0.019 | 0.778 | 0.043 | 0.524 | -0.010 | 0.886 | -0.033 | 0.628 | 0.001 | 0.988 | -0.030 | 0.655 |
| Sequential mean t3 DIS | -0.075 | 0.267 | 0.035 | 0.608 | -0.008 | 0.905 | -0.039 | 0.571 | -0.041 | 0.544 | 0.013 | 0.844 | 0.011 | 0.873 |
| Sequential mean cat1 t3 DIS | -0.046 | 0.495 | 0.025 | 0.714 | -0.004 | 0.955 | 0.088 | 0.195 | 0.106 | 0.120 | 0.016 | 0.810 | 0.038 | 0.576 |
| Sequential mean cat2 t3 DIS | 0.038 | 0.580 | 0.005 | 0.944 | 0.035 | 0.607 | -0.076 | 0.265 | -0.107 | 0.117 | -0.063 | 0.357 | -0.025 | 0.717 |
| Cumulative mean t1 DIS | -0.031 | 0.652 | -0.056 | 0.408 | 0.108 | 0.111 | 0.139 | 0.040 | 0.110 | 0.106 | 0.038 | 0.576 | 0.065 | 0.339 |
| Cumulative mean t2 DIS | 0.001 | 0.991 | 0.036 | 0.598 | 0.033 | 0.632 | 0.039 | 0.566 | 0.030 | 0.662 | 0.010 | 0.878 | 0.024 | 0.722 |
| Cumulative mean t3 DIS | 0.087 | 0.199 | 0.093 | 0.170 | 0.004 | 0.955 | -0.021 | 0.758 | -0.027 | 0.696 | 0.048 | 0.480 | 0.071 | 0.300 |

t1=VF test (animals); t2= VF test (jobs); t3= Switching VF test (sports/fruits); cat1=1$^{st}$ category in VF switching task (sports); cat2=2$^{nd}$ category in VF switching task (fruits); DIS=semantic system *DISCO*

**Supplementary Table S5: Correlations of all semantic verbal fluency features and significantly predicted EF scores (working memory and inhibition tests)**

| | NBN - Errors | | NVLT - Correct | | NVLT - Errors | | NVLT - Difference correct-false | | STROOP - Naming interference | | STROOP - Process time | |
|---|---|---|---|---|---|---|---|---|---|---|---|---|
| | r | p | r | p | r | p | r | p | r | p | r | p |
| Correct words t1+t2+t3 | 0.039 | 0.563 | 0.051 | 0.456 | -0.001 | 0.992 | 0.033 | 0.631 | -0.221 | 0.001 | -0.194 | 0.004 |
| Correct words t1 | 0.008 | 0.910 | 0.024 | 0.727 | -0.059 | 0.388 | 0.074 | 0.275 | -0.152 | 0.025 | -0.160 | 0.018 |
| Correct words t2 | 0.080 | 0.242 | 0.026 | 0.701 | 0.073 | 0.284 | -0.057 | 0.403 | -0.212 | 0.002 | -0.101 | 0.136 |
| Correct words t3 | 0.004 | 0.959 | 0.092 | 0.176 | 0.000 | 1.000 | 0.058 | 0.394 | -0.163 | 0.016 | -0.218 | 0.001 |
| Repetition error t1 | 0.107 | 0.114 | 0.009 | 0.899 | 0.135 | 0.046 | -0.131 | 0.054 | -0.039 | 0.564 | -0.011 | 0.877 |
| Repetition error t2 | 0.054 | 0.431 | -0.074 | 0.277 | 0.161 | 0.017 | -0.209 | 0.002 | -0.076 | 0.265 | -0.040 | 0.552 |
| Repetition error t3 | -0.008 | 0.907 | -0.089 | 0.190 | -0.032 | 0.641 | -0.024 | 0.721 | 0.024 | 0.725 | 0.003 | 0.966 |
| Category error t3 | -0.007 | 0.919 | 0.022 | 0.741 | 0.047 | 0.490 | -0.033 | 0.626 | 0.025 | 0.717 | -0.060 | 0.380 |
| Latency mean t1 | -0.030 | 0.656 | -0.072 | 0.293 | 0.083 | 0.221 | -0.129 | 0.057 | 0.074 | 0.280 | 0.076 | 0.267 |
| Latency mean t2 | -0.062 | 0.360 | -0.014 | 0.840 | -0.035 | 0.605 | 0.027 | 0.694 | 0.155 | 0.022 | 0.031 | 0.649 |
| Latency mean t3 | 0.008 | 0.907 | -0.090 | 0.187 | 0.037 | 0.586 | -0.094 | 0.167 | 0.159 | 0.019 | 0.179 | 0.008 |
| Latencies 1st quarter t1 | -0.027 | 0.697 | -0.048 | 0.479 | -0.027 | 0.688 | -0.003 | 0.967 | 0.185 | 0.006 | 0.144 | 0.033 |
| Latencies 1st quarter t2 | -0.032 | 0.634 | 0.046 | 0.500 | -0.052 | 0.445 | 0.081 | 0.231 | 0.165 | 0.015 | 0.110 | 0.104 |
| Latencies 1st quarter t3 | -0.008 | 0.906 | -0.052 | 0.445 | 0.035 | 0.608 | -0.068 | 0.317 | 0.119 | 0.081 | 0.124 | 0.069 |
| Latencies 2nd quarter t1 | 0.004 | 0.954 | 0.044 | 0.515 | 0.098 | 0.150 | -0.071 | 0.300 | -0.012 | 0.855 | 0.017 | 0.804 |
| Latencies 2nd quarter t2 | -0.074 | 0.277 | -0.018 | 0.790 | -0.092 | 0.176 | 0.081 | 0.232 | 0.107 | 0.116 | 0.027 | 0.692 |
| Latencies 2nd quarter t3 | 0.113 | 0.095 | -0.024 | 0.723 | 0.064 | 0.344 | -0.080 | 0.239 | 0.172 | 0.011 | 0.194 | 0.004 |
| Latencies 3rd quarter t1 | -0.033 | 0.624 | -0.079 | 0.244 | 0.047 | 0.494 | -0.097 | 0.153 | 0.033 | 0.632 | 0.064 | 0.345 |
| Latencies 3rd quarter t2 | 0.040 | 0.557 | -0.032 | 0.640 | -0.004 | 0.950 | -0.016 | 0.817 | 0.021 | 0.762 | -0.035 | 0.605 |
| Latencies 3rd quarter t3 | -0.085 | 0.210 | 0.003 | 0.963 | -0.022 | 0.751 | 0.024 | 0.727 | 0.068 | 0.318 | 0.011 | 0.871 |
| Latencies 4th quarter t1 | -0.056 | 0.408 | -0.083 | 0.222 | 0.065 | 0.341 | -0.118 | 0.083 | 0.048 | 0.484 | 0.055 | 0.421 |
| Latencies 4th quarter t2 | -0.016 | 0.816 | -0.010 | 0.882 | 0.031 | 0.652 | -0.037 | 0.583 | 0.104 | 0.127 | -0.017 | 0.804 |
| Latencies 4th quarter t3 | 0.052 | 0.442 | -0.052 | 0.441 | -0.025 | 0.715 | -0.008 | 0.906 | -0.063 | 0.353 | -0.003 | 0.960 |
| Latency difference t1 | -0.054 | 0.423 | -0.080 | 0.242 | 0.067 | 0.325 | -0.118 | 0.083 | 0.034 | 0.617 | 0.044 | 0.516 |
| Latency difference t2 | -0.012 | 0.859 | -0.016 | 0.819 | 0.037 | 0.587 | -0.047 | 0.489 | 0.085 | 0.212 | -0.030 | 0.661 |
| Latency difference t3 | 0.053 | 0.435 | -0.049 | 0.475 | -0.028 | 0.685 | -0.003 | 0.966 | -0.072 | 0.287 | -0.013 | 0.850 |
| Sequential mean t1 | -0.192 | 0.005 | -0.026 | 0.699 | 0.017 | 0.806 | -0.033 | 0.623 | -0.016 | 0.814 | -0.004 | 0.959 |
| Sequential mean t2 | 0.045 | 0.505 | -0.104 | 0.127 | 0.007 | 0.921 | -0.072 | 0.288 | -0.181 | 0.008 | -0.030 | 0.660 |
| Sequential mean t3 | -0.033 | 0.629 | -0.017 | 0.799 | -0.037 | 0.592 | 0.026 | 0.705 | -0.027 | 0.688 | -0.029 | 0.668 |
| Cumulative mean t1 | -0.014 | 0.842 | 0.015 | 0.825 | -0.052 | 0.441 | 0.062 | 0.359 | -0.175 | 0.010 | -0.170 | 0.012 |
| Cumulative mean t2 | 0.053 | 0.433 | -0.039 | 0.568 | 0.014 | 0.832 | -0.039 | 0.565 | -0.184 | 0.006 | -0.113 | 0.096 |
| Cumulative mean t3 | 0.001 | 0.992 | 0.037 | 0.583 | -0.041 | 0.547 | 0.065 | 0.340 | -0.181 | 0.007 | -0.204 | 0.002 |
| Sequential mean cat1 t3 | -0.114 | 0.094 | 0.037 | 0.582 | 0.018 | 0.792 | 0.006 | 0.935 | -0.120 | 0.078 | -0.127 | 0.061 |
| Sequential mean cat2 t3 | -0.015 | 0.829 | 0.014 | 0.838 | -0.031 | 0.652 | 0.040 | 0.560 | 0.020 | 0.765 | -0.056 | 0.412 |
| Switch coefficient | -0.048 | 0.483 | 0.038 | 0.576 | 0.001 | 0.990 | 0.023 | 0.734 | 0.104 | 0.126 | 0.006 | 0.924 |
| Sequential mean t1 DIS | -0.087 | 0.201 | 0.004 | 0.954 | 0.017 | 0.802 | -0.015 | 0.829 | -0.064 | 0.349 | -0.015 | 0.821 |
| Sequential mean t2 DIS | 0.083 | 0.223 | 0.082 | 0.226 | 0.095 | 0.161 | -0.044 | 0.517 | -0.169 | 0.012 | 0.002 | 0.980 |
| Sequential mean t3 DIS | 0.033 | 0.631 | 0.013 | 0.847 | -0.052 | 0.447 | 0.061 | 0.374 | -0.010 | 0.880 | -0.009 | 0.896 |
| Sequential mean cat1 t3 DIS | -0.081 | 0.234 | 0.097 | 0.153 | 0.121 | 0.075 | -0.060 | 0.374 | -0.147 | 0.030 | -0.065 | 0.341 |
| Sequential mean cat2 t3 DIS | -0.128 | 0.060 | 0.048 | 0.478 | -0.083 | 0.220 | 0.115 | 0.091 | 0.015 | 0.825 | -0.021 | 0.763 |
| Cumulative mean t1 DIS | -0.004 | 0.953 | -0.059 | 0.384 | 0.045 | 0.505 | -0.083 | 0.221 | 0.014 | 0.840 | -0.001 | 0.992 |
| Cumulative mean t2 DIS | -0.036 | 0.597 | 0.075 | 0.270 | -0.074 | 0.279 | 0.122 | 0.073 | -0.073 | 0.282 | -0.008 | 0.902 |
| Cumulative mean t3 DIS | -0.123 | 0.069 | -0.016 | 0.819 | -0.082 | 0.227 | 0.073 | 0.284 | -0.105 | 0.121 | -0.058 | 0.393 |

t1=VF test (animals); t2= VF test (jobs); t3= Switching VF test (sports/fruits); cat1=1st category in VF switching task (sports); cat2=2nd category in VF switching task (fruits); DIS=semantic system *DISCO*

## Supplementary Table S6: Correlations of all semantic verbal fluency features and significantly predicted EF scores (attention tests)

| | WAF-G - rt unimodal | | WAF-G - Missed items crossmodal | | WAF-G - False alarm crossmodal | | WAF- rt crossmodal | | WAF-R - rt correct announced | | WAF-R - rt short SOA | | WAF-R - rt long SOA | |
|---|---|---|---|---|---|---|---|---|---|---|---|---|---|---|
| | r | p | r | p | r | p | r | p | r | p | r | p | r | p |
| Correct words t1+t2+t3 | -0.019 | 0.783 | -0.124 | 0.068 | -0.067 | 0.326 | -0.073 | 0.286 | 0.007 | 0.922 | -0.067 | 0.321 | -0.035 | 0.602 |
| Correct words t1 | -0.026 | 0.705 | -0.180 | 0.008 | -0.166 | 0.014 | -0.095 | 0.163 | -0.021 | 0.759 | -0.058 | 0.390 | -0.047 | 0.490 |
| Correct words t2 | 0.047 | 0.490 | 0.002 | 0.972 | 0.075 | 0.271 | 0.016 | 0.819 | 0.024 | 0.719 | -0.046 | 0.496 | -0.005 | 0.947 |
| Correct words t3 | -0.086 | 0.204 | -0.092 | 0.175 | -0.031 | 0.654 | -0.096 | 0.158 | 0.026 | 0.704 | -0.054 | 0.425 | -0.027 | 0.687 |
| Repetition error t1 | -0.035 | 0.604 | 0.043 | 0.528 | 0.019 | 0.776 | -0.052 | 0.447 | -0.092 | 0.174 | -0.089 | 0.190 | -0.095 | 0.164 |
| Repetition error t2 | 0.045 | 0.512 | 0.027 | 0.695 | 0.120 | 0.077 | -0.027 | 0.697 | -0.006 | 0.926 | -0.037 | 0.590 | 0.008 | 0.905 |
| Repetition error t3 | -0.118 | 0.083 | -0.029 | 0.674 | -0.013 | 0.852 | -0.093 | 0.171 | 0.011 | 0.875 | -0.004 | 0.952 | 0.031 | 0.652 |
| Category error t3 | -0.026 | 0.701 | 0.025 | 0.718 | 0.073 | 0.286 | -0.034 | 0.620 | -0.041 | 0.545 | -0.040 | 0.560 | -0.041 | 0.543 |
| Latency mean t1 | 0.019 | 0.783 | 0.121 | 0.075 | 0.063 | 0.352 | 0.056 | 0.408 | -0.069 | 0.312 | -0.028 | 0.686 | -0.038 | 0.581 |
| Latency mean t2 | -0.057 | 0.402 | 0.005 | 0.942 | -0.081 | 0.235 | -0.045 | 0.505 | -0.009 | 0.899 | 0.055 | 0.420 | 0.003 | 0.969 |
| Latency mean t3 | 0.070 | 0.302 | 0.097 | 0.155 | 0.041 | 0.544 | 0.096 | 0.157 | -0.019 | 0.775 | 0.035 | 0.603 | 0.007 | 0.919 |
| Latencies 1st quarter t1 | 0.057 | 0.402 | 0.061 | 0.372 | 0.008 | 0.901 | -0.023 | 0.730 | -0.015 | 0.825 | 0.012 | 0.859 | 0.028 | 0.677 |
| Latencies 1st quarter t2 | -0.043 | 0.530 | 0.047 | 0.491 | -0.042 | 0.534 | -0.002 | 0.980 | 0.013 | 0.850 | 0.044 | 0.515 | 0.037 | 0.590 |
| Latencies 1st quarter t3 | 0.091 | 0.182 | 0.001 | 0.991 | 0.030 | 0.656 | 0.127 | 0.061 | -0.108 | 0.112 | -0.027 | 0.695 | -0.069 | 0.311 |
| Latencies 2nd quarter t1 | 0.063 | 0.356 | 0.038 | 0.574 | 0.015 | 0.830 | 0.141 | 0.038 | -0.029 | 0.672 | 0.013 | 0.850 | -0.007 | 0.916 |
| Latencies 2nd quarter t2 | -0.031 | 0.645 | -0.097 | 0.152 | -0.065 | 0.340 | 0.020 | 0.771 | -0.062 | 0.365 | 0.009 | 0.893 | -0.043 | 0.529 |
| Latencies 2nd quarter t3 | 0.022 | 0.752 | 0.084 | 0.215 | 0.049 | 0.476 | 0.046 | 0.499 | 0.043 | 0.531 | 0.088 | 0.194 | 0.053 | 0.440 |
| Latencies 3rd quarter t1 | 0.019 | 0.784 | 0.098 | 0.151 | 0.079 | 0.246 | 0.028 | 0.683 | -0.045 | 0.507 | -0.013 | 0.843 | -0.039 | 0.571 |
| Latencies 3rd quarter t2 | -0.056 | 0.409 | 0.033 | 0.628 | -0.036 | 0.594 | -0.085 | 0.211 | 0.024 | 0.729 | 0.038 | 0.572 | -0.005 | 0.939 |
| Latencies 3rd quarter t3 | -0.010 | 0.883 | 0.003 | 0.959 | -0.045 | 0.506 | -0.003 | 0.963 | -0.042 | 0.536 | -0.010 | 0.882 | -0.008 | 0.903 |
| Latencies 4th quarter t1 | -0.050 | 0.459 | 0.163 | 0.016 | 0.122 | 0.071 | 0.086 | 0.209 | 0.028 | 0.677 | 0.020 | 0.764 | 0.027 | 0.688 |
| Latencies 4th quarter t2 | -0.007 | 0.922 | 0.079 | 0.245 | 0.045 | 0.512 | -0.097 | 0.155 | 0.058 | 0.396 | 0.066 | 0.334 | 0.015 | 0.823 |
| Latencies 4th quarter t3 | -0.032 | 0.636 | 0.062 | 0.364 | -0.008 | 0.903 | -0.052 | 0.446 | 0.012 | 0.860 | -0.005 | 0.940 | 0.007 | 0.923 |
| Latency difference t1 | -0.055 | 0.421 | 0.158 | 0.019 | 0.122 | 0.072 | 0.087 | 0.199 | 0.030 | 0.665 | 0.020 | 0.774 | 0.025 | 0.710 |
| Latency difference t2 | -0.002 | 0.980 | 0.074 | 0.277 | 0.050 | 0.464 | -0.097 | 0.154 | 0.057 | 0.406 | 0.061 | 0.371 | 0.011 | 0.872 |
| Latency difference t3 | -0.039 | 0.565 | 0.062 | 0.362 | -0.011 | 0.876 | -0.062 | 0.364 | 0.020 | 0.766 | -0.003 | 0.964 | 0.012 | 0.861 |
| Sequential mean t1 | 0.048 | 0.483 | -0.008 | 0.903 | -0.121 | 0.074 | -0.004 | 0.951 | 0.007 | 0.913 | 0.050 | 0.459 | 0.037 | 0.588 |
| Sequential mean t2 | 0.041 | 0.545 | 0.010 | 0.886 | 0.018 | 0.795 | -0.057 | 0.406 | -0.002 | 0.982 | -0.004 | 0.952 | 0.000 | 0.995 |
| Sequential mean t3 | -0.103 | 0.130 | -0.039 | 0.564 | -0.107 | 0.116 | -0.077 | 0.256 | -0.048 | 0.482 | -0.058 | 0.396 | -0.061 | 0.371 |
| Cumulative mean t1 | -0.019 | 0.776 | -0.094 | 0.169 | -0.176 | 0.009 | -0.036 | 0.593 | 0.059 | 0.383 | 0.055 | 0.415 | 0.059 | 0.387 |
| Cumulative mean t2 | 0.032 | 0.639 | 0.078 | 0.249 | 0.038 | 0.579 | -0.013 | 0.845 | 0.002 | 0.976 | 0.010 | 0.883 | -0.013 | 0.848 |
| Cumulative mean t3 | -0.061 | 0.371 | -0.030 | 0.662 | -0.089 | 0.189 | -0.049 | 0.475 | -0.007 | 0.913 | -0.039 | 0.570 | -0.062 | 0.362 |
| Sequential mean cat1 t3 | -0.066 | 0.331 | -0.114 | 0.094 | -0.065 | 0.339 | -0.048 | 0.479 | -0.063 | 0.355 | -0.057 | 0.405 | -0.083 | 0.222 |
| Sequential mean cat2 t3 | -0.167 | 0.014 | -0.127 | 0.060 | -0.025 | 0.714 | -0.108 | 0.110 | 0.013 | 0.853 | -0.029 | 0.665 | -0.046 | 0.496 |
| Switch coefficient | -0.074 | 0.279 | 0.058 | 0.397 | 0.053 | 0.436 | -0.014 | 0.839 | 0.021 | 0.760 | 0.027 | 0.695 | 0.015 | 0.822 |
| Sequential mean t1 DIS | 0.012 | 0.865 | 0.007 | 0.921 | -0.042 | 0.536 | -0.006 | 0.933 | 0.035 | 0.607 | 0.033 | 0.628 | 0.021 | 0.759 |
| Sequential mean t2 DIS | 0.080 | 0.237 | -0.002 | 0.982 | 0.020 | 0.766 | 0.111 | 0.101 | 0.036 | 0.593 | 0.044 | 0.522 | 0.068 | 0.321 |
| Sequential mean t3 DIS | -0.021 | 0.756 | 0.016 | 0.815 | -0.046 | 0.498 | 0.018 | 0.792 | 0.149 | 0.028 | 0.121 | 0.076 | 0.101 | 0.138 |
| Sequential mean cat1 t3 DIS | -0.019 | 0.783 | -0.030 | 0.656 | -0.025 | 0.715 | -0.065 | 0.343 | -0.064 | 0.345 | -0.067 | 0.326 | -0.108 | 0.112 |
| Sequential mean cat2 t3 DIS | -0.073 | 0.285 | -0.052 | 0.443 | -0.044 | 0.515 | 0.058 | 0.393 | -0.035 | 0.609 | -0.059 | 0.384 | -0.057 | 0.404 |
| Cumulative mean t1 DIS | -0.007 | 0.919 | 0.125 | 0.065 | 0.173 | 0.010 | 0.048 | 0.482 | 0.072 | 0.287 | 0.049 | 0.475 | 0.076 | 0.266 |
| Cumulative mean t2 DIS | 0.048 | 0.480 | 0.007 | 0.916 | -0.047 | 0.494 | 0.063 | 0.357 | 0.101 | 0.135 | 0.105 | 0.123 | 0.102 | 0.135 |
| Cumulative mean t3 DIS | -0.073 | 0.280 | -0.066 | 0.332 | -0.102 | 0.133 | -0.007 | 0.922 | -0.039 | 0.563 | -0.059 | 0.385 | -0.073 | 0.286 |

t1=VF test (animals); t2= VF test (jobs); t3= Switching VF test (sports/fruits); cat1=1st category in VF switching task (sports); cat2=2nd category in VF switching task (fruits); DIS=semantic system *DISCO*
